# Adsorption of Nonionic Surfactants (Nonylphenols) on Sandstone Rock via Alcoholic Micellar Solution

**DOI:** 10.1021/acs.langmuir.4c01628

**Published:** 2024-09-05

**Authors:** Valdivino Francisco dos Santos Borges, Mayra Kerolly Sales Monteiro, Ernani Dias da Silva Filho, Dennys Correia da Silva, José Luís Cardozo Fonseca, Alcides O. Wanderley Neto, Tiago Pinheiro Braga

**Affiliations:** † Institute of Chemistry, Postgraduate Program in Chemical - PPGQ, Federal University of Rio Grande do Norte (UFRN), Senador Salgado Filho Avenue, Lagoa Nova District, Natal 59078-970, RN, Brazil; ‡ Laboratory of Environmental and Applied Electrochemistry - LEAA, Postgraduate Program in Chemical Engineering - PPGEQ, Federal University of Rio Grande do Norte (UFRN), Senador Salgado Filho Avenue, Lagoa Nova District, Natal 59078-970, RN,Brazil; § Department of Petroleum Engineering, Federal University of Rio Grande do Norte (UFRN), Senador Salgado Filho Avenue, Lagoa Nova District, Natal 59078-970, RN, Brazil

## Abstract

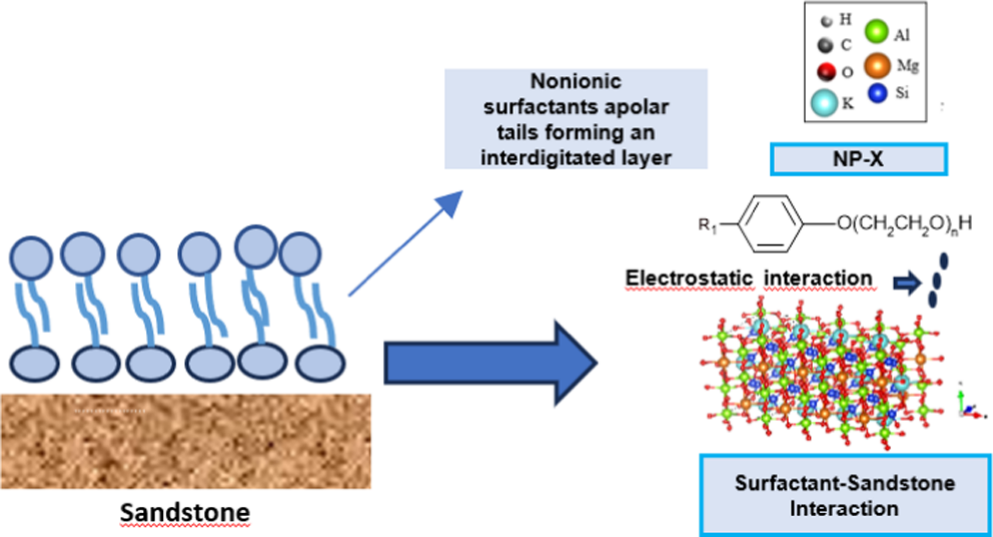

The adsorption of surfactants on rock surfaces can modify their hydrophobicity, surface charge, and other important properties that govern advanced oil recovery processes, such as decreasing the interfacial tension between water and oil and increasing permeability. Generally, the need to control and/or reduce surfactant adsorption on reservoir rock surfaces has been a challenging task in enhanced oil recovery (EOR) methods, as it directly impacts the project’s economics. This requires a comprehensive study and understanding of the adsorption mechanism on rocks. This work investigates the adsorption process of nonionic surfactants from the family of ethoxylated nonylphenols in alcoholic micellar solutions on sandstone rock surfaces. The systems used in the experiments consisted of NP 9.5EO, NP 11EO, and NP 15EO, butanol as an amphiphilic solvent, and a saline solution (2% KCl) as the aqueous phase. The experiments were conducted according to the Scheffé network and showed an adsorption efficiency of 66.89% for NP-15EO, 67.15% for NP-11EO, and 70.60% for NP-9.5EO, thus proving that the higher the degree of ethoxylation of nonylphenols, the lower the adsorption capacity. Point F was chosen as the optimum point since this point remained constant during the experiments, besides being a water-rich region with low butanol content. The sandstone exhibited oil-favorable wettability, which after treatment resulted in wettability inversion, with a decrease in the contact angle with water, a factor that can increase oil recovery. Adsorption isotherm modeling was also performed to investigate the adsorption mechanism. All adsorption tests followed and best fit the Redlich–Peterson isotherm, showing that the adsorption process occurs in monolayers and multilayers. The experimental methodology also involves analyses of mineralogy, morphology, thermal stability, and surface charge of the sandstone rock.

## Introduction

Ethylene oxide (EO) surfactants belong to the group of nonionic surfactants that have been extensively studied recently due to their wide industrial applications such as oil recovery, flotation, flocculation-dispersion, and lubrication. These molecules are amphiphilic, meaning they are composed of hydrophilic parts (polar head) and hydrophobic parts (nonpolar tail).^1−6^ Due to this duality, surfactants can alter the properties of a multiphase system, such as reducing the oil–water interfacial tension, changing the surface wettability of a rock from oil-wet to water-wet. These properties explain the importance of introducing surfactants into a hydrocarbon reservoir to improve oil recovery.^7−12^

Their wide applicability in industry depends on the nature of the layers adsorbed on solids and/or gases. In this sense, it is important to understand the orientation of molecules in the adsorbed layers, elucidate the mechanisms of adsorption involved, and determine the most suitable composition in the synthesis of formulations, which can make the process more economical.^1,5^ Several authors have studied how surfactant adsorption alters wettability and its effect on oil recovery in different types of reservoir rocks.^13,14^ Very high adsorption is economically unfeasible as it requires a large amount of surfactants.^15−19^ Austad et al. observed^20^ that the test with the highest oil recovery occurred with a lower water–oil contact angle on the surfactant substrate (<80°). Wettability alteration refers to the change in the contact angle at the oil–water-rock three-phase contact line. Therefore, it is essential to know the surfactant–substrate interactions responsible for adsorption and to obtain adsorption isotherms that relate the equilibrium adsorption of the surfactant at the solid–liquid interface to the equilibrium concentration of the surfactant in solution at a specific temperature.^21−28^ Thus, the isotherms are necessary to determine the amount of surfactant loss in the adsorbent.

In a previous work, Araújo et al.^7^ studied the adsorption of three different surfactants from the ethoxylated nonylphenol class on sandstone rocks both statically and in flow. However, there is a relentless search for the study of adsorption behavior, wettability alteration, and consequently surfactant selection, especially of the various nonylphenols, which remains unprecedented, due to the various EOR processes that require different strategies to optimize surfactant selection, and this choice depends heavily on the conditions of the oil reservoir.^8,29^ In this research, the adsorption mechanism of three surfactants from the ethoxylated nonylphenols class (9.5EO, 11EO, and 15EO) on sandstone rocks via alcoholic micellar solution (AMS) was evaluated, as well as the applied isotherm models to mathematically understand how the adsorption of these surfactants occurred, elucidating the main parameters responsible for altering the medium wettability.

A Scheffé system model was used to perform experiments at different mass ratios. For process optimization, point *F* was chosen, composed of 87.5% aqueous phase (*X*_ap_), 2.5% butanol (*X*_but_), and 10% surfactant (*X*_s_), as this point remained constant during duplicate experiments, besides presenting a single-phase region, rich in water and with low alcohol content, and with an adsorption efficiency (AE) of 90% for NP 9.5EO and 85% for NP 11EO and 15EO. After choosing the optimal point of the experiments, physicochemical characterizations of the adsorbent were performed. Mineralogical and elemental analyses of sandstone samples were carried out using X-ray diffraction (XRD) and X-ray fluorescence (XRF), respectively. Scanning Electron Microscopy (SEM), Fourier Transform Infrared Spectroscopy (FTIR) and Thermogravimetric Analysis (TG/DTG) are found in Supporting Information and were used to observe changes in the morphology and spectrum of the surfactant, the thermal stability on the rock surface before and after adsorption, respectively.

## Experimental Section

### Materials

The surfactants, alcohol, and salt utilized in this study are listed in Table 1. The nonionic surfactants are characterized by ethoxylated groups in their structure were supplied by the Brazilian company Oxiteno S.A. These nonylphenol ethoxylates, denoted as NP-*x*, originate from petroleum refining process involving the alkylation of phenol with isomeric nonane groups under acidic catalysis. Subsequently, ethylene oxide chains are added to the molecule’s hydroxyl group through an ethoxylation process, converting it into a nonionic surfactant. The general formula for nonionic surfactants, the methods used for contact angle measurements and the treated samples are shown in Figures S1, S2 and S3 of the Supporting Information.

**Table 1:** Chemical Compounds Used in This Research

reagents	name	chemical formula	provider	specification (*x*)	purity (%)
alcohol	butanol	C_4_H_10_O	vetec		99.4
salt	potassium chloride	KCl	chemical dynamics		99.0
surfactant	nonylphenol 9.5EO	C_15_H_23_(OC_2_H_4_)_9.5_OH	Oxiteno S.A.	9.5	99.5
	nonylphenol 11 EO	C_15_H_23_(OC_2_H_4_)_11_OH	Oxiteno S.A.	11	99.5
	nonylphenol 15 EO	C_15_H_23_(OC_2_H_4_)_15_OH	Oxiteno S.A.	15	99.5

These nonionic surfactants result from the reaction of nonylphenol with a variable number of ethylene oxide (EO) molecules. The sandstone rocks, obtained from the Botucatu Formation (Rio Grande do Norte - Brazil), were crushed in ball mills for 24 h. Then, the calcination process was carried out to remove the moisture and organic materials contained in the pores of the rocks and increase their permeability. The samples were dried in a muffle furnace for a period of 6 h at *T* = 250 °C, with a heating rate of 10 °C per minute, under an air atmosphere. After drying, they were sieved under mechanical agitation, using a series of sieves ranging from 48 to 100 mesh for 10 min.

### Methods

#### Finite Bath Adsorption Tests

For the finite bath adsorption tests, 0.05 g of Botucatu sandstone, as the adsorbent, with a particle size of 100 mesh (0.149 mm), were weighed. Then, 10 mL of alcoholic micellar solution (AMS) of known initial concentration (*C*_0_) was added. The samples were placed in a thermostatic bath (digital water bath, model NT249, Novatecnica) with internal and external circulation, for 4 h at a temperature of 40 ± 1 °C. After reaching the desired contact time, the sample was centrifuged (6000 rpm) to separate the rock grains from the supernatant.

The quantification of the surfactant content in the AMS was performed using a procedure similar to that used in previous Works,^7,30^ employing the spectroscopy technique using a UV–vis spectrophotometer (Genesys 10 UV–vis, Thermo Electron Corporation) at a wavelength of 274 nm for the three surfactants used.

Over time, the concentration of the adsorbate decreased, indicating that part of the surfactant was adsorbed onto the rock. The adsorption capacity was then calculated based on the surfactant concentrations determined by eq 1
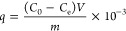
1Where *q* (mg/g-rock) denotes the static adsorption extent of the surfactant on the rock surface, *C*_0_ (mg/L or ppm) represents the preadsorption surfactant concentration, *C* (mg/L or ppm) shows the postadsorption surfactant concentration, *V* (mL) denotes the surfactant solution volume, and m (g) is the mass of the powdered rock sample.^31^

The values of *C*_0_ varied from 2.5, 3.75, 5.0, 6.25, 7.5 to 10.0% of surfactants. The batch tests were conducted at a temperature of 40 °C, following the methodology proposed by Araújo et al.,^7^ which is consistent with the average temperature of Brazilian oil reservoirs. After conducting the experiments, the curve of the amount of solute on the adsorbent (*q*_e_) versus concentration (*C*_e_) could be constructed.

#### Adsorption Isotherm Model

An adsorption isotherm represents the amount of material adsorbed per unit mass of the adsorbent and is determined as a function of the concentration in solution at a constant temperature. It is the most widely used method for representing the equilibrium states of an adsorption system.^32,33^ This means that adsorption isotherm models are needed to predict the adsorption behavior of the surfactant at a given concentration.^33−35^ The isotherm models used in this study are described below.

##### Langmuir Adsorption Isotherm

The Langmuir equation was one of the pioneers in proposing an explanation for the phenomenon of adsorption on a uniform, simple and nonporous surface.^32,36^ It is currently applied to various types of adsorbent. Langmuir’s adsorption isotherm assumes an empirical model of monolayer adsorption based on kinetic principles without accumulation in equilibrium conditions. The adsorption of molecules occurs under a fixed and defined number of sites (homogeneous adsorption), all of which have the capacity to adsorb only one molecule at a time, and without it interacting with the others adsorbed by neighboring sites, i.e., there is no lateral interaction between the adsorbed molecules. This means that each site has the same amount of energy and is capable of forming just one surfactant molecule. The nonlinear form of the Langmuir isotherm can be written as (eq 2)
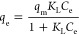
2Where *q*_e_ (mg/g) is the adsorbed amount of the surfactant at an equilibrium condition, *q*_m_ (mg/g) is the maximum amount of the adsorbed surfactant, *C*_e_ (mg/L) is the equilibrium concentration of the adsorbate, and *K*_L_ (L/mg) is the Langmuir constant.

##### Freundlich Adsorption Isotherm

The Freundlich isotherm assumes that adsorption of the adsorbate occurs on a heterogeneous surface in multilayers.^37^ This model considers the solid to be heterogeneous, while applying an exponential decay to the energy distribution of the adsorbed sites.^32^ The Freundlich isotherm takes the form of eq 3
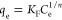
3Where *K*_F_ (L/mg) is the adsorption capacity and 1/*n* is the surface heterogeneity. A adsorção é considerada favorável quando 0 < 1/*n* < 1, desfavorável quando 1/*n* > 1 e irreversível quando 1/*n* = 1.

##### Temkin Adsorption Isotherm

The Temkin isotherm is a two-parameter equation that takes into account adsorbent–adsorbate interactions and adsorption is characterized by a uniform distribution of binding energies.^32^ This adsorption model assumes that, due to some interactions between the adsorbent and the adsorbate, the heat of adsorption should reduce linearly when coating the solid surface with surfactant molecules.^38^ As a result, this adsorption isotherm is usually presented in the form of the following linear equation (eq 4)
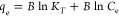
4In this equation, the parameter *q*_e_ is the amount of adsorption at equilibrium (mg/g), *B* (*RT*/*b*) and *K_T_* (L/mg) are the Temkin adsorption isotherm constants or binding equilibrium constants, *C*_e_ is the equilibrium concentration (mg/L), and the parameter *T* is the absolute temperature in Kelvin (*K*), *R* is the universal gas constant (8.314 J/mol K) and the parameter *b* is a constant that is related to the heat of adsorption.

##### Redlich–Peterson Adsorption Isotherm

The Redlich–Peterson isotherm is used to represent adsorption equilibrium over a wide range of concentrations. É uma isoterma de adsorção de três parâmetros que combina elementos das isotermas de Langmuir e Freundlich.^36^ This model has an exponential function in the denominator and linear dependence in the numerator. Because of this versatility, it is applied to both homogeneous and heterogeneous systems.^33,37^
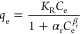
5Where *K*_R_ (L/g) is the constant of the Redlich–Peterson isotherm, β_r_ is the exponent that varies between 0 and 1, and α_r_ is a constant in L/mg. The model approximates the Langmuir isotherm at low concentrations (β_r_ ≅ 1) and the Freundlich isotherm at high concentrations (β_r_ ≅ 0).

### Ternary Diagrams and Calibration Curve

**Figure 1:**
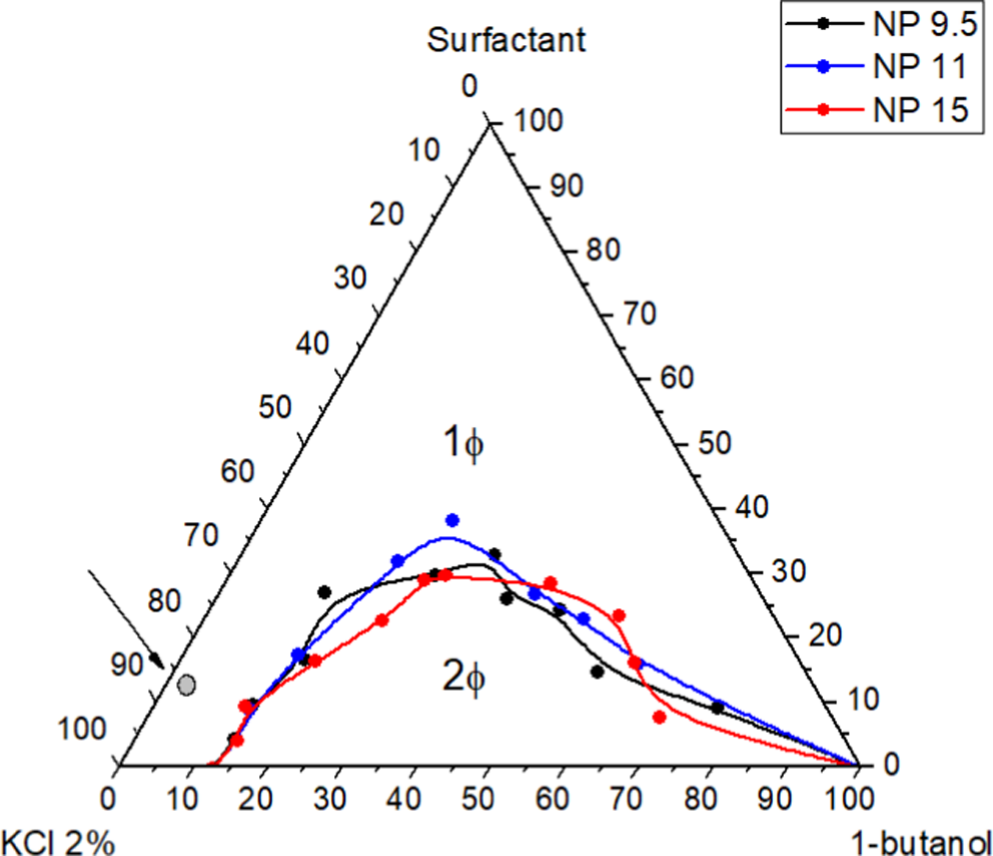
Ternary diagram of systems composed of 1-butanol, brine (KCl 2%) in surfactants with different degrees of ethoxylation. The arrow indicates the composition: 87.5% brine, 2.5% 1-butanol, and 10% surfactant.

Three AMS systems were obtained using the ternary diagram, using a brine solution, potassium chloride (KCl 2%) as the aqueous phase, butanol and ethoxylated nonylphenol with 9.5, 11, and 15 ethoxy groups (9.5EO, 11EO and 15EO), respectively, as surfactants, uma vez que, este sistema ainda não foi utilizado em pesquisas anteriores, trazendo desta forma, formulações inéditas para uma possível aplicação industrial. A conventional oil phase was not used, since alcohol as a cosurfactant can fulfill the function of an oil, as it is a predominantly apolar molecule. This makes this formulation less harmful to the environment. In previous work, researchers studied the effect of the presence of crude oil on the adsorption of anionic-nonionic surfactants in sandstone and the influence of the partitioning of these surfactants on the quantification of adsorption and found that the adsorption values were much higher when crude oil was present compared to the adsorption values when crude oil was absent.^11^Figure 1 shows the obtained ternary diagram; This figure also shows the Scheffé lattice (A-J) used in this work and listed in the Supporting Information in Table S1, in order to compare the different adsorption efficiencies in a given parameter.

Upon observing the diagram, two regions are noticed: a single-phase region (1ϕ) characterized by the solubilization of the three constituents (water, butanol, and surfactant) and a biphasic region (2ϕ) characterized by an insufficient amount of surfactant to form micelles, resulting in two phases (water and butanol). In this work, the formulation of the single-phase system (1ϕ) was chosen. Volumetric titration analysis was used to delimit the area of this region.

For this purpose, five mixtures of different compositions of surfactants and cosurfactants (*X*_s_ + *X*_c_ = 2.0 g, 10–50% by weight of surfactant) were titrated drop by drop with the aqueous phase (brine) until there was a phase change: from clear to cloudy. The same procedure was carried out with five mixtures of different compositions of surfactant and aqueous phase (*X*_s_ + *X*_a_ = 2.0 g, 10–50% by weight of surfactant) using the cosurfactant as a titrant. This totaled 10 trials of each surfactant.

The calibration curve was obtained before the adsorption tests. For this purpose, the spectrum was analyzed from 190 to 400 nm to determine the optimum wavelength for the NP-*x* surfactants. The solution was diluted to obtain different concentrations of surfactants and the absorbance (A) was measured at the wavelength found using the Lambert–Beer equation (eq 6), which relates the absorbance of an unknown sample to the concentration of the solute present.
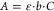
6where ε is the absorptivity at 274 nm, *b* is the optical path length, and *C* is the solute concentration.

The wavelength with the highest absorbance obtained was 274 nm for the three surfactants, a value close to that found in the literature using nonionic ethoxylated nonylphenols. From there, the absorbance values were converted into solute concentration present in the solvent through the calibration curve and linear regression equations (*y* = *ax* + *b*), where *y* represents the absorbance and *x* represents the concentration of the solution.

The data measured and obtained for the construction of the calibration curve are listed in Table S2 of the Supporting Information, which shows the linear regression equations and Pearson’s correlation coefficients (*R*^2^), through which it was possible to obtain a good fit to the experimental data, thus quantifying the content of surfactants present after the adsorption tests. Measurement of the contact angle with the aqueous phase

In this work, the DSA100 droplet analysis optical system was used to determine the dynamic contact angle of surfactants and rock, as shown in Figure S2 in Supporting Information.

The procedures carried out in determining the contact angle for the sandstone rock were based on the methodology used by Li et al.,.^1−3,19^ After obtaining the plugs, these were sectioned into discs (tablets) with a diameter of 39 mm and a thickness of 5.5 mm. Since the plugs are wetted during the cutting of the tablets, they were dried in an oven at 100 °C until complete evaporation of water, a process that lasted 1 h.

Subsequently, the samples were placed in a desiccator until reaching room temperature, and then soaked with Ubarana oil (from Rio Grande do Norte - Brazil) and taken back to the oven, subjected to a temperature of 50 °C for 48 h, to remove the excess oil surrounding them, and finally allowed to dry at room temperature.

After drying, the tablets were immersed in alcoholic micellar systems (AMS) for 1 h. After this time, it was observed that the surface did not present a wetted appearance. This drying process lasted 5 days.

Considering adsorption efficiency as the response variable, Statistica 7.0 software was used to estimate the influence of the factors and their interactions. Analysis of variance was also carried out on the model and the response surface was generated. Figure S4 in the Supporting Information represents the Pareto chart for the data obtained with 95% confidence for nonylphenols.

The proximity of the equation to the experimental data can be assessed by the graph of predicted values versus observed values in Figure S5 in the Supporting Information. The response surfaces of the adsorption efficiencies (AE%) for each model were obtained, as shown in Figure S6 in the Supporting Information, and it was observed that the efficiency increased with increasing surfactant concentration.

It can be seen that all the systems showed an adsorption percentage greater than >80%. Point *F* (87.5% by weight of *X*_a_, 2.5% by weight of *X*_but_ and 10% by weight of *X*_s_) was chosen as the ideal AMS due to its high aqueous phase content and low alcohol content, which makes it less toxic to the environment.

The samples to be tested were fixed on the test platform of a Kruss goniometer, and the syringe was installed. The needle position and droplet shape were controlled by the control panel; contact angles were measured, and the final result was read by the instrument. Table S4 found in the Supporting Information shows the measurements of the contact angle between the sandstone and the titration with distilled water before and after treatment with AMS and the percentage reduction compared to the untreated rock.

### Rock Characterization

X-ray diffraction (XRD) and X-ray fluorescence (XRF) of the sandstone powder were performed to analyze the mineralogical and elemental characteristics of the rock, respectively. The peaks of the XRD patterns were acquired using a Bruker D2Phaser diffractometer equipped with a Lynxeye detector and copper radiation (Cu Kα, λ = 1.54 Å) with a Ni filter, 10 mA current, 30 kV voltage. The collected data was analyzed over a wide range of Bragg angle 2θ°, ranging from 10 to 80°, with a step size of 0.02°, acquisition time of 0.1 s, and temperature of 298 K. XRF was performed on a Bruker S2 Ranger instrument, with Pd or Ag anodes, maximum power of 50 W, 50 kV, 2 mA current, and coupled to a Silicon X Flash detector.

FTIR analysis was performed using an IRAffinity-1 apparatus manufactured by Shimadzu, coupled with an HATR MIRacle module with a ZnSe prism, and the wavenumber of the spectrum ranging from 400 to 4000 cm^–1^. The signal-to-noise ratio and resolution were 30,000:1 for a 1 min scan at a resolution of 4 cm^–1^ at 2100 cm^–1^.

#### Zeta potential (ζ) Measurements

For zeta potential measurements, 40 mL of sandstone aqueous solution was used to determine the electrokinetic mobility, μE, using a Zeta-Meter System 3.0+ (Zeta-Meter Inc.). The zeta potential at equilibrium (ζ) was calculated using the Smoluchowski equation, according to the methodology performed by Filho et al.^39^ (eq 7)
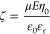
7Where η_0_ is the viscosity of the continuous phase, ε_0_ is the electric permittivity of vacuum, and ε_r_ is the relative permittivity of the continuous phase. The average of 10 measurements was reported as the zeta potential (ζ) of the sample.

## Results and Discussion

### Finite Bath Adsorption and the Scheffé Network

The adsorption efficiencies obtained for the AMS of NP-9.5EO, NP-11EO, and NP-15EO are observed in Tables 2, 3, and 4, respectively.

**Table 2:** Percentage of Adsorption Efficiency According to the Scheffé Network for the NP 9.5EO Solution

	samples	conc. (%)	AE* (%)
	A	1.70	31.80
	B	1.64	34.34
	C	1.20	51.94
	D	1.17	81.26
NP 9.5EO	E	1.46	76.66
	F	0.99	90.04
	G	0.87	82.63
	H	0.72	80.73
	I	0.62	83.52
	J	0.51	93.13
	(***x̅***)		70.60

**Table 3:** Percentage of Adsorption Efficiency According to the Scheffé Network for the NP 11EO Solution

	samples	conc. (%)	AE (%)
	A	1.70	31.87
	B	1.60	35.96
	C	0.14	94.35
	D	1.55	75.10
NP 11EO	E	1.42	77.32
	F	1.48	85.18
	G	1.50	69.92
	H	1.47	60.68
	I	1.46	61.06
	J	1.50	80.03
	(***x̅***)		67.15

**Table 4:** Percentage of Adsorption Efficiency According to the Scheffé Network for the NP 15EO Solution^a^

	samples	conc. (%)	AE (%)
	A	1.43	42.92
	B	1.42	42.92
	C	0.61	75.68
	D	1.45	76.78
NP 15EO	E	1.46	72.60
	F	1.47	85.25
	G	1.46	70.88
	H	1.44	60.38
	I	1.45	61.30
	J	1.46	80.15
	(***x̅***)		66.89

a Duplicate Mean, Conc.: concentration, AE: adsorption efficiency, *x̅*: arithmetic mean.

According to the results obtained, the structure of the hydrophobic and hydrophilic units of the surfactants affected the degree of adsorption and the alteration of wettability as seen in the previous section. Adsorption increased for surfactants with fewer hydrophilic groups, with an average of 66.89% for NP-15EO, 67.15% for NP-11EO, and 70.60% for NP-9.5EO.

**Figure 2:**
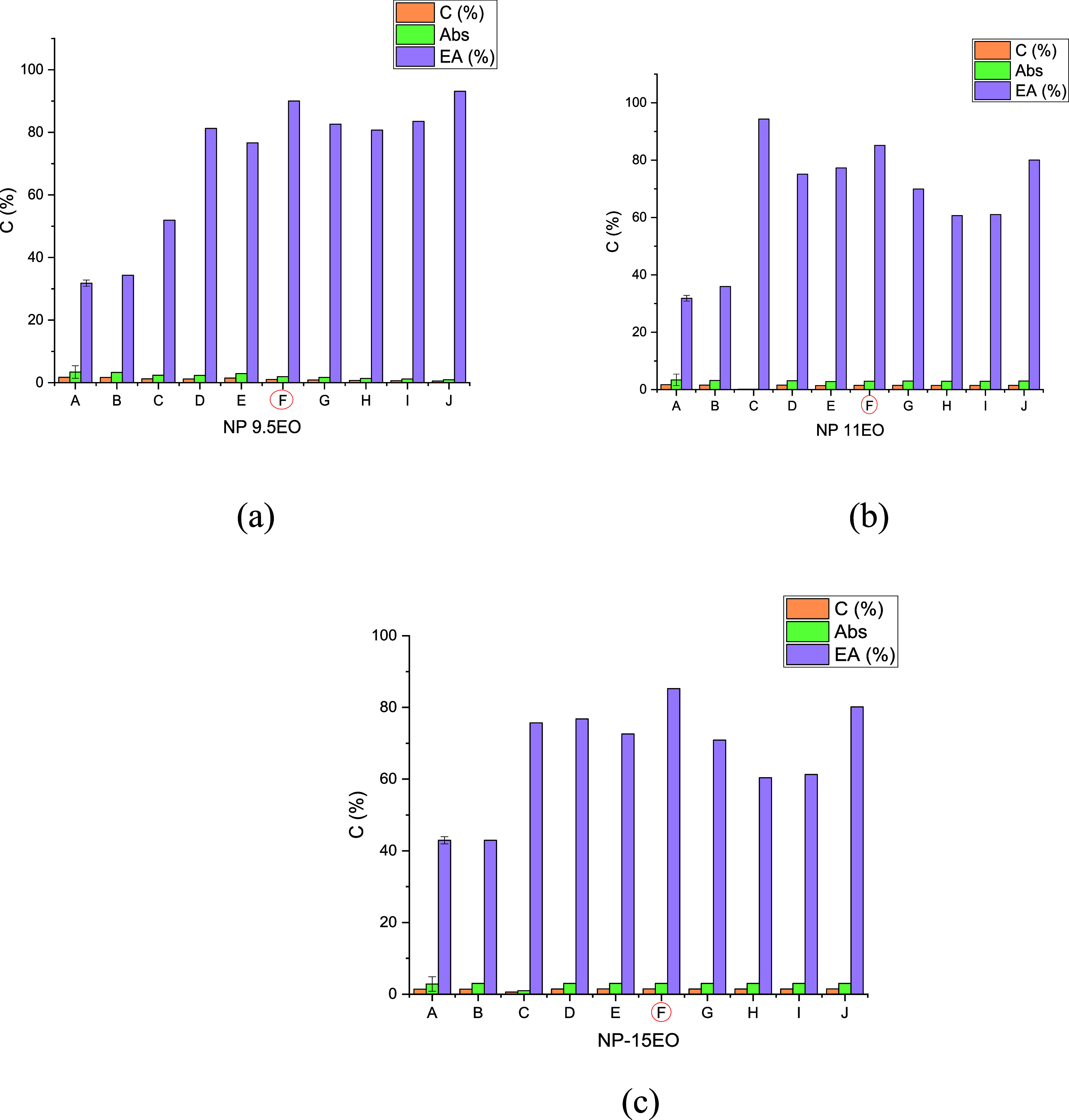
Comparison of adsorption plateaus for the surfactants (a) NP-9.5EO, (b) NP-11EO and (c) NP-15EO, highlighting the optimum point chosen (Point F).

Since adsorption occurs as micelles rather than individual surfactant molecules, an increase in adsorption was observed for the more hydrophobic surfactants, attributed to the increase in micelle size. The graphs in Figure 2a–c illustrate these results, with emphasis on point *F* of the Scheffé network, chosen as the optimum point for all the nonylphenols studied, since this point remained constant during the duplicate experiments.

The arithmetic mean of the adsorption efficiencies confirms that the degree of ethoxylation had a strong influence on the adsorption process. The greatest adsorption occurs when the surfactant has the smallest ethoxy group, since it prefers to adsorb to the rock and is not completely soluble in the aqueous phase. In the case of NP-9.5EO, adsorption reached 90% at the optimum point.

The arithmetic mean of the adsorption efficiencies confirms that the degree of ethoxylation strongly influences the adsorption process. This is due to the fact that adsorption is higher when the surfactant has a smaller ethoxy group, as it prefers to adsorb onto the rock rather than being totally soluble in the aqueous phase. In the case of NP-9.5EO, for example, adsorption reached 90% at the optimum point. This fact is also related to its polarity, the surfactant with the smallest ethoxy group is less polar than surfactants with larger ethoxy groups, which is what happens with the NP-9.5EO surfactant, which due to its low polarity tends to adsorb onto the sandstone which is a hydrophobic surface, while NP-15EO and NP-11EO tend to remain in the aqueous phase due to their high polarities.

**Figure 3:**
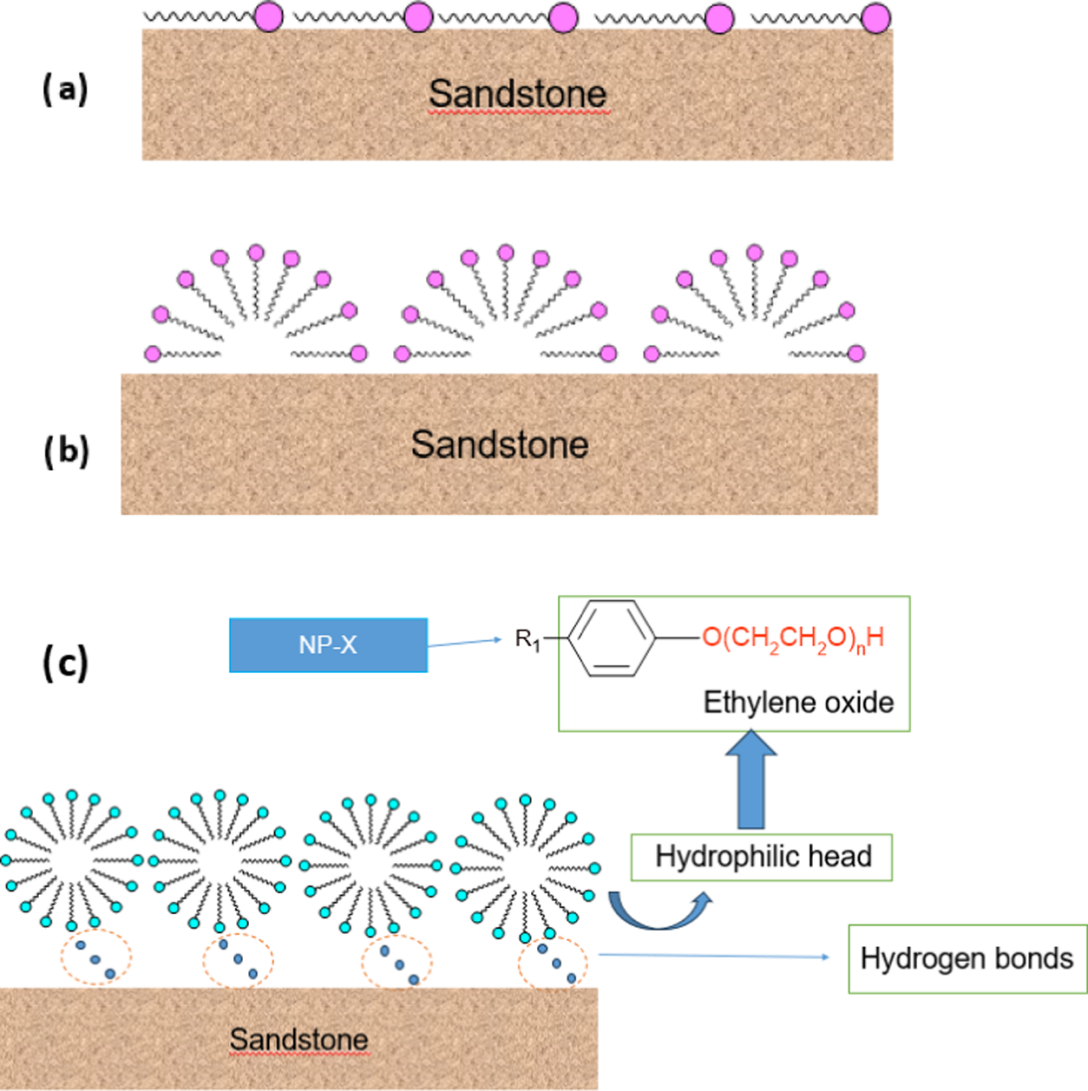
Proposed mechanism for the adsorption of nonionic surfactants on sandstone rock (a–b). Adsorption of nonylphenols (NP-*x*) in the form of micelles on the sandstone surface (c).

Figure 3a–c show the proposed mechanism for the adsorption of nonylphenols on the solid surface of sandstone.

Initially, few surfactant molecules are adsorbed on the surface (Figure 3a). The surfactant molecules are adsorbed on the surface (Figure 3a), which is mainly due to adsorption by Wan der Waals interactions, mainly due to the hydrophobic part of the surfactant, hydrogen bonds and hydrophilic interactions.

Then, as the surfactant concentration increases, the surface is saturated by a monolayer (Figure 3b). As the sandstone has a hydrophilic surface, the monomers are organized with their heads facing the surface, forming hemimicelles and linked by hydrogen bonds between the ethoxylated groups of the surfactants and the silane groups of the rock. Adsorption generally takes place in the form of irregular micellar aggregates, thus increasing the hydrophilicity of the surface and consequently altering the surface’s wettability properties and ability to interact with other molecules, as seen in Figure 3. Hydrogen interactions between the polar groups of the surfactant and the hydrophilic surfaces of the sandstone played a crucial role.

Considering adsorption efficiency as the response variable, Statistica 7.0 software was used to estimate the influence of factors and their interactions. Analysis of variance of the model was also performed, and the response surface was generated. Figure S4 represents the Pareto chart for the data obtained with 95% confidence for nonylphenols, as presented in the Supporting Information.

### Adsorption Isotherms

**Figure 4:**
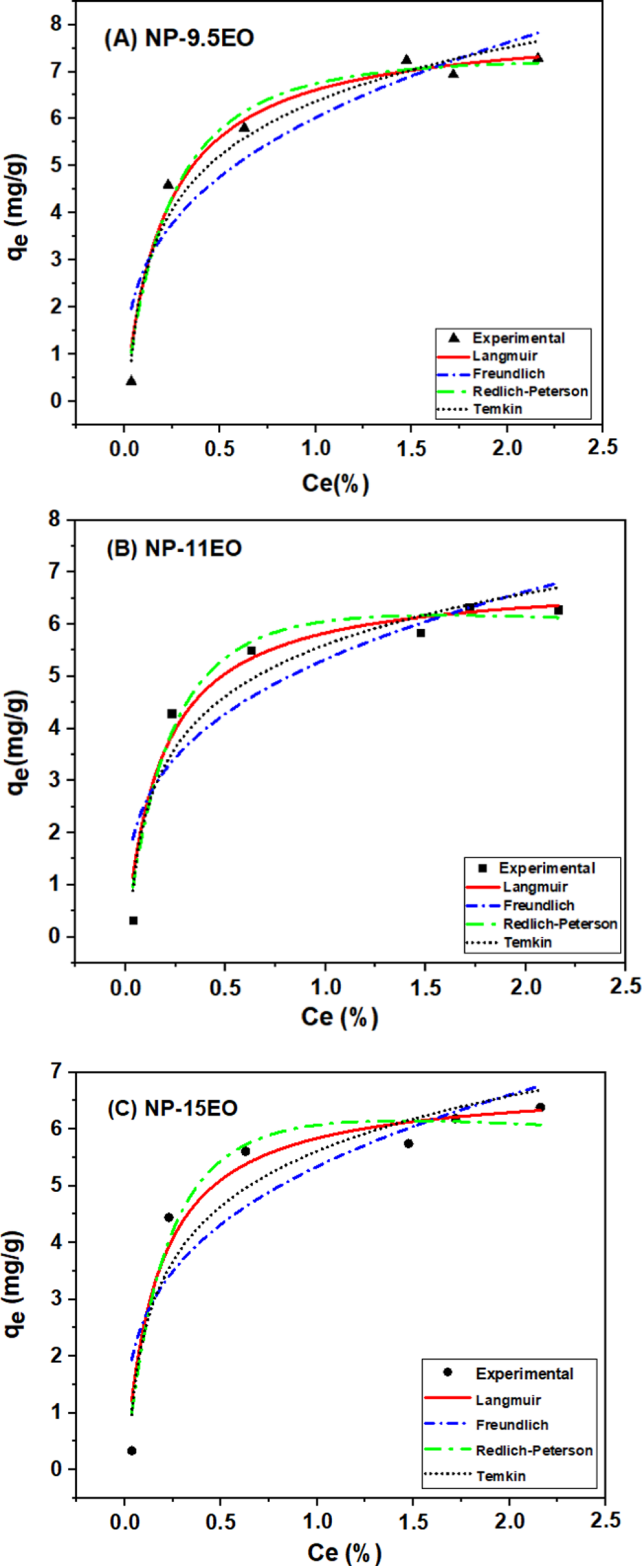
Adsorption isotherms for AMS NP-9.5EO, NP-11EO, and NP-15EO (*T* = 40 °C).

Representing the equilibrium relationship between the adsorbate (surfactant) and the adsorbent (sandstone), adsorption isotherms at 40 °C were studied for A MSNP-9.5EO, NP-11EO and NP-15EO, represented in Figure 4a–c, respectively. Four adsorption isotherms (Langmuir, Freundlich, Temkin, and Redlich–Peterson) were fitted to the resulting data, and their adsorption parameters were calculated using eqs 2–5, as proposed by Araújo et al.,^7^ The coefficient of determination (*R*^2^) was used to assess the performance of the adsorption models.

According to the graphs, the degree of ethoxylation had a significant influence on the equilibrium adsorption capacity. It was observed that the adsorption of surfactants increases with decreasing hydrophilicity, meaning that the lower the number of EO (ethylene oxide) groups in the surfactant, the higher its adsorption capacity. The validity of the model is determined according to the *R*^2^ value, the closer it is to 1, the better the experiment values fit the applied model, as shown in Table S3 Supporting Information.

Observing the parameter values obtained for each model along with the fitting equation and the respective coefficient of correlation (*R*^2^), the Redlich–Peterson model showed the best fit, with *R*^2^ values closest to 1 compared to the other models.

The Redlich–Peterson isotherm is used to represent adsorption equilibrium over a wide range of concentrations and can be applied to both homogeneous and heterogeneous systems. Due to its versatility, this model can combine characteristics of the Langmuir and Freundlich models, where adsorption can occur in monolayers or multilayers, respectively. According to the data in Tables 2, 3, and 4 this phenomenon is observed for the adsorption of nonylphenols: NP-9.5EO, NP-11EO, and NP-15EO.

It was observed that as the degree of ethoxylation decreased, the adsorption capacity increased, making adsorption favorable. This behavior was also observed by Araújo et al.,^7^ using nonylphenols NP-10EO, NP-40EO and NP-100EO.

### Measurement of Contact Angle and Wettability

This section presents the results of contact angle measurements on Botucatu sandstone tablets in both natural and treated states with alcoholic micellar systems (AMS).

The mechanism of treatment with AMS is reported as an interaction between surfactant monomers involved in the micelle and the negatively charged organic carboxylates in petroleum, suggesting the formation of ions that remove the carboxylate and form a water-wetted surface.^1,3^ One factor associated with wettability reversal may be the 2% salt (KCl) added to the aqueous phase, which increases the surfactant’s solubility, making the alcoholic micellar solution more polar.

After adsorption on the rock, it becomes more polarized due to the presence of chloride ions, aiding in water droplet spreading due to the chemical interaction between the deposited liquid and the rock surface.

Another factor that may have contributed to the different contact angle values is the amount of ethoxy groups present in each surfactant, as the molecule becomes more polar with more ethoxy groups. Thus, the surfactant with more ethoxy groups prefers to stay in the aqueous phase rather than adsorbed on the rock.

**Figure 5:**
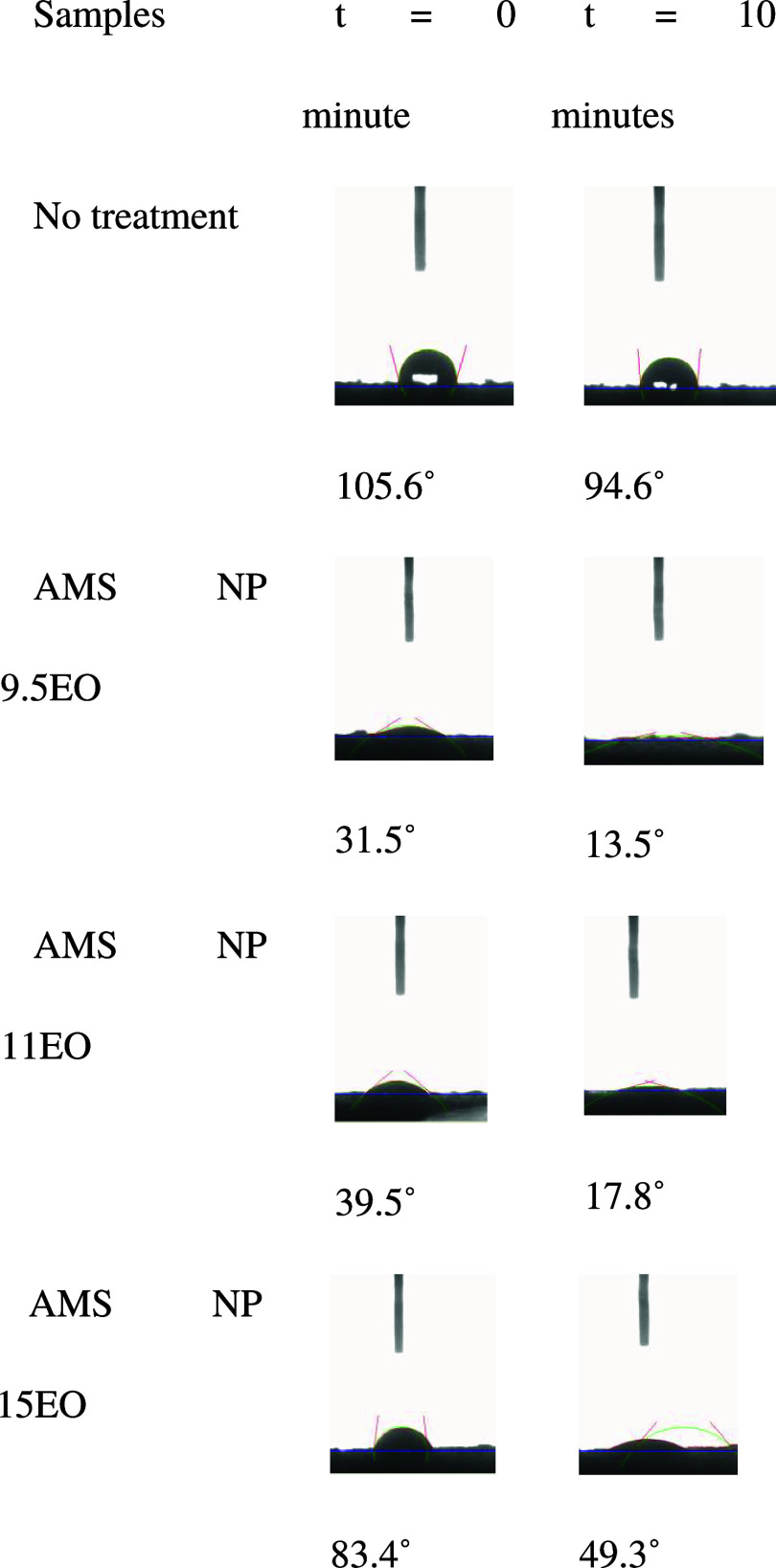
Evolution of Botucatu sandstone wetting upon contact with oil, after being wetted by a drop of distilled water, during the first 10 min of contact with AMS.

In this case, the surfactant acted as a rock surface modifier, reversing its wettability from oil-wet to water-wet. Figure 5 depicts this behavior with and without treatment during the first 10 min of analysis.

The high contact angle value for untreated Botucatu sandstone is likely due to the high surface tension and hydrophobicity of the rock-oil surface. As the rock surface is oil-wet, it tends to occupy all its pores, hindering the interaction of the water droplet.

Oil (an apolar fluid) is more attracted to the rock due to its hydrophobic surface, making it more difficult to extract from a reservoir, while water, being a polar fluid, hardly interacts with it. However, in the case of untreated rock, as time passes, gravitational forces push the droplet downward, leading to a minimal reduction in the contact angle to 94.6° within 10 min.

For the treated tablets, it is observed that the AMS adsorb to their surface, improving wetting behavior. This phenomenon is shown in the initial measurement (at *t* = 0 s) for all systems, where the low contact angle values already indicate a change in wettability.

This proves that the composition for the formation of AMS reduces electrostatic repulsions between the heads of ethoxylated surfactant molecules, favoring micellar packing, allowing better interfacial coverage, resulting in lower contact angle values, and consequently, greater wetting.

### Zeta Potential, Diffractogram, and X-ray Fluorescence

**Figure 6:**
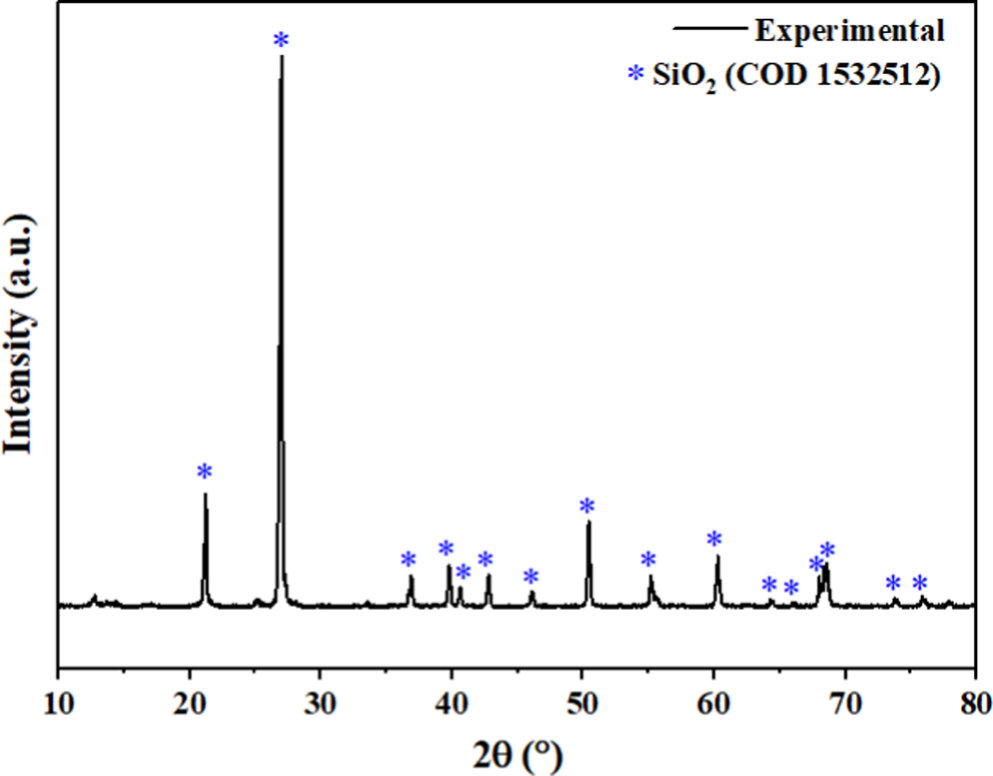
Diffraction pattern of the Botucatu sandstone sample.

It is observed that the positive potential of the sandstone is possibly due to its nature; the value of +29.0 mV indicates an excess of positive charges. DRX and FRX tests were conducted to analyze the constituents of the Sandstone samples, as shown in Figure 6 and Table 5, respectively.

**Table 5:** Results of X-ray Fluorescence Chemical Analysis of Botucatu Sandstone

	components	(%)
Botucatu sandstone	SiO_2_	77. 08
	Al_2_O_3_	17. 06
	Na_2_O	1. 7
	MgO	1. 6
	Fe_2_O_3_	1.03
	CaO	0.57
	TiO_2_	0.35
	SO_3_	0. 21
	Cl	0.21
	K_2_O	0.19
	ZrO_2_	0.03

According to the diffraction pattern of the sandstone sample, variations in the position and relative intensities of peaks are observed, as well as changes in the number of peaks, demonstrating the structural complexity of this mineral.

Quartz is the mineral representing the group of tectosilicates, appearing with greater intensity in the diffraction pattern, indicating that the majority of the sandstone used in this study is composed of this mineral. It is represented by the main peak with an interplanar distance *d* = 3.30 Å and angle 2θ = 27.01°, a secondary peak with *d* = 4.18 Å and angle 2θ = 21.22°, and a tertiary peak with *d* = 1.80 Å and angle 2θ = 50.60°.

The pretreated sandstone sample shows peaks corresponding to quartz combined with the COD/PDF 1532512 database, respectively in the PANalytical High Score software. Regarding the crystallite size, the sandstone presented a diameter of 44.09 nm.

The results of the FRX show a significant amount of Si in the sandstone, confirming the results of the DRX.

The quartz (SiO_2_) is the predominant oxide in the analyzed sample, averaging 77% by weight of the samples. Alumina (Al_2_O_3_) occurs in significant amounts, averaging 17% by weight. These data show that over 90% of the material is composed of the oxides SiO_2_ and Al_2_O_3_, comprising almost all of the material.

These results with smaller quantities of sodium, magnesium, iron, calcium, titanium, and potassium are compatible with the minerals quartz, Illite, and calcite, identified by XRD. Therefore, the sandstone mineralogy contains a significant amount of quartz along with clay content and some impurities.

It can be observed that there were no changes in the crystalline structure of the rock after the adsorption tests with the AMS.

### FTIR Spectroscopy

**Figure 7:**
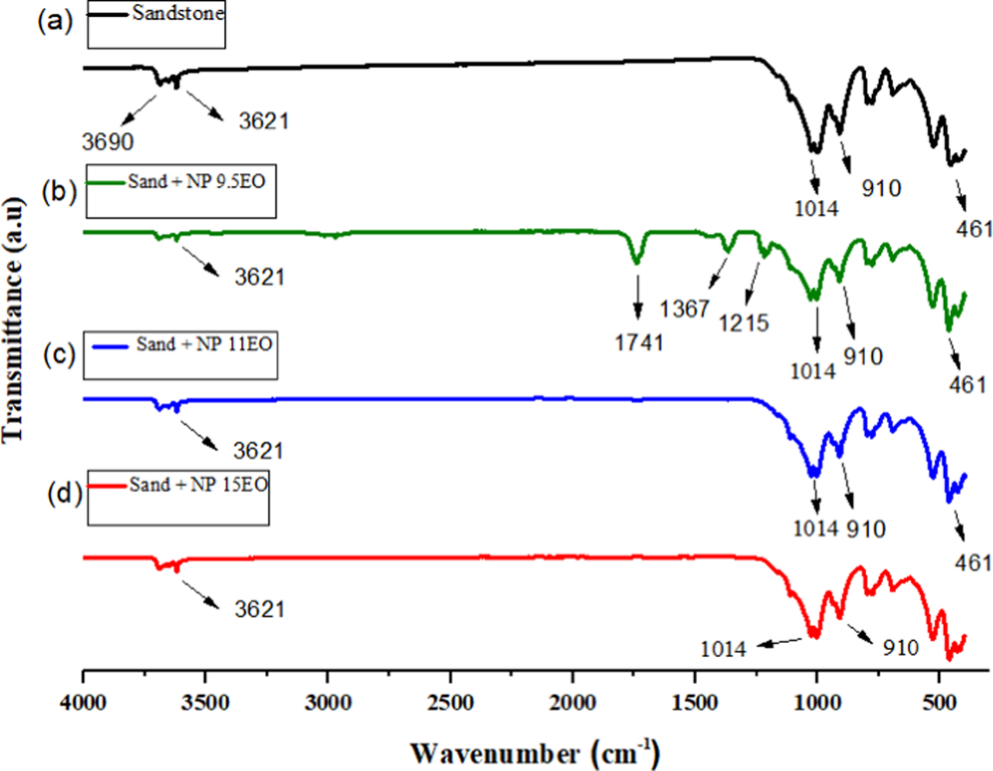
FTIR spectrum for (a) pure sandstone, (b) sandstone adsorbed with SMA NP-9.5EO, (c) sandstone adsorbed with AMS NP-11EO, and (d) sandstone adsorbed with AMS NP-15EO.

Figure 7a shows for the pure sandstone and Figure 7b–d shows for the sandstone adsorbed with AMS NP-9.5EO, NP-11EO, and NP-15EO, respectively.

As observed in the Figures above, the FTIR spectrum of pure sandstone exhibits bands at 461 and 1014 cm^–1^ corresponding to the vibrational stretching region for the symmetric (υSi–Os) and asymmetric (υSi-Oas) Si–O groups, respectively, indicating that the sandstone contains pure silica in its main composition. Additionally, bands at 910 cm^–1^ corresponding to quartz grains are observed. The sandstone sample also shows bands at 3621 to 3690 cm^–1^ corresponding to the symmetric (υC-Hs) and asymmetric (υC-Has) stretching of the −CH_2_ groups, respectively.

After the adsorption of the surfactants, a slight reduction in the bands at 3690–3621 cm^–1^ was observed in all spectra. A band at 1741 cm^–1^ was observed in the absorption spectrum of Figure 7b of the sandstone + NP-9.5EO, likely originating from the vibrational stretching of the OH group of water molecules during the surfactant adsorption.

Complementary analyses of the mineralogy and morphology of the sandstone rock and studies of the thermal stability of the adsorbed nonylphenols in order to emphasize the effect of mineralogy on the adsorption characteristics of these surfactants can be found in the Supporting Information in Figures S7, S8, S9, S10, S11 and S12.

## Conclusions

In this study, the adsorption mechanism of nonylphenols in different concentrations of alcoholic micellar solutions in sandstone rock was investigated through static adsorption experiments, adsorption isotherm models, and analyses of ζ potential, XRD, XRF, FTIR, TG/DTG, and SEM. From the experimental data it was observed that the adsorption efficiency increased for surfactants with fewer hydrophilic groups, averaging 66.89% for NP-15EO, 67.15% for NP-11EO, and 70.60% for NP-9.5EO. The chosen optimum point comprised 87.5% aqueous phase, 2.5% butanol, and 10% surfactant. It covered the monophasic region of the ternary diagram and achieved a adsorption efficiency of 90% for NP 9.5EO and 85% for NP 11 and 15EO. Based on correlation coefficients (*R*^2^), the Redlich–Peterson isotherm was the best model to describ the equilibrium adsorption behavior of nonylphenols on sandstone surfaces, where adsorption was observed on both monolayer and multilayer surfaces. The sandstone was characterized as a hydrophobic surface with a high contact angle value (105.6°) with distilled water; however, after treatment, there was a significant reduction in the contact angle for all systems. Therefore, the systems were effective in altering the wettability of the rock. The maximum contact angle value on the rock surface after treatment was in the order: AMS NP 15EO > AMS NP 11EO > AMS NP 9.5EO. The positively charged surface of the sandstone (ζ = +29.35 mV) did not significantly influence the adsorption process, as there was an increase in hydrophobic interactions between the nonylphenol’s apolar tails, thus playing a governing role over electrostatic interaction forces. Observations from XRD, XRF, FTIR, TG/DTG, and SEM confirm the proposed adsorption behavior of nonylphenols on sandstone rock surfaces.

## Supporting Information Available

General formula for a nonionic surfactant; Scheffé network used to study the adsorption of nonylphenols in sandstone; linear adjustment of the calibration curve; DSA 100 optical droplet shape analyzer; Sandstone rock tablets coated with oil and after the final treatment with nonylphenol alcoholic micellar systems; graphs of predicted values by the equation versus observed values; response surface graphs for the adsorption efficiency; parameters obtained from the adsorption isotherm models; contact angle measurements between the sandstone and titration with distilled water before and after treatment with AMS; FTIR spectra for the nonylphenols; thermal stability analysis of sandstone rock (PDF)
