# Supplementary material for: Adsorption of Nonionic Surfactants (Nonylphenols) on Sandstone Rock via Alcoholic Micellar Solution
Source: Langmuir. 2024 Sep 5;40(37):19430–40. doi: 10.1021/acs.langmuir.4c01628 (PMC11411723; doi:10.1021/acs.langmuir.4c01628)
Supplement: Supplementary file 1 — la4c01628_si_001.pdf [file la4c01628_si_001.pdf]

Supporting Information: Adsorption of nonionic surfactants (nonylphenols) on sandstone rock via alcoholic micellar solution.

Valdivino Francisco dos Santos Borges[a], Mayra Kerolly Sales Monteiro[b], Ernani Dias da Silva Filho[c], Dennys Correia da Silva[c], José Luís Cardozo Fonseca[a], Alcides O. Wanderley Neto[a] and Tiago Pinheiro Braga[a]*

[a] *Institute of Chemistry, Postgraduate Program in Chemical - PPGQ, Federal University of Rio Grande do Norte (UFRN), 59078-970 Senador Salgado Filho Avenue, Lagoa Nova district, Natal-RN, Brazil*

[b] *Laboratory of Environmental and Applied Electrochemistry - LEAA, Postgraduate Program in Chemical Engineering - PPGEQ, Federal University of Rio Grande do Norte (UFRN), 59078-970 Senador Salgado Filho Avenue, Lagoa Nova district, Natal-RN, Brazil*

[c] *Department of Petroleum Engineering, Federal University of Rio Grande do Norte (UFRN), 59078-970 Senador Salgado Filho Avenue, Lagoa Nova district, Natal-RN, Brazil*

[*] *Email: valdivino.santos@ifro.edu.br*

Figure: S1: General formula for a nonionic surfactant. The hydrophilic part is represented by repeated units of ethylene oxide, and the hydrophobic part by a nonylphenol group. Where: n = degree of ethoxylation.

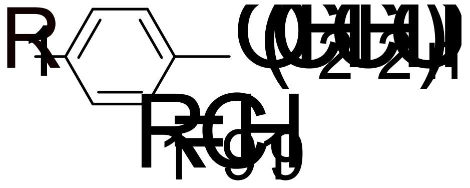

Table S1: Scheffé network used to study the adsorption of nonylphenols in sandstone.

Ensaio	(X_{AP}) (%)	(X_{but}) (%)	(X_S) (%)
A	95	2,5	2,5
B	91,25	6,25	2,5
C	87,5	10	2,5
D	91,25	2,5	6,25
E	87,5	6,25	6,25
F	87,5	2,5	10
G	90	5	5
H	92,33	3,75	3,75
I	88,75	7,5	3,75
J	88,75	3,75	7,5

* Duplicate Mean, X_{AP}: Aqueous Phase, X_{But}: Butanol, X_S: Surfactant, Conc.: Concentration, AE: Adsorption Efficiency, $\bar{x}$: Arithmetic Mean.

Table S2: Linear adjustment of the calibration curve for nonylphenols NP 9.5EO, NP 11EO, and NP 15EO.

Surfactant	Equation (y = ax + b)	Pearson Correlation (R2)
NP-9,5EO	y = 4.833x + 0.058	0.995
NP-11EO	y = 6.250x + 0.002	0.998
NP-15EO	y = 5.160x + 0.003	0.995

Figure S2: (a) DSA 100 optical droplet shape analyzer (b) Contact angle test.

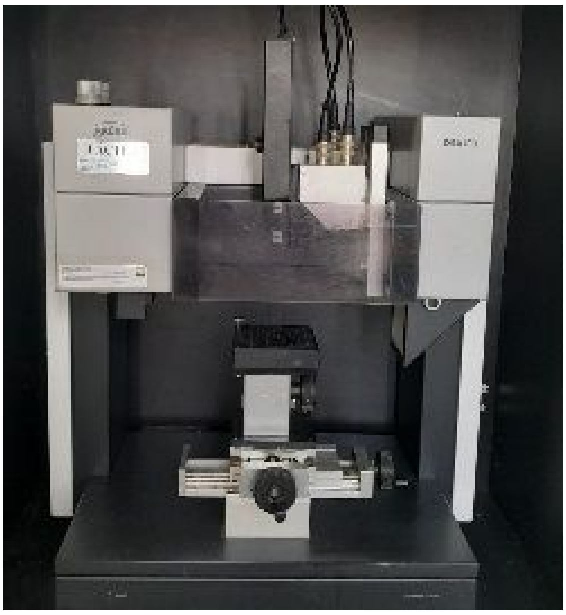

(a)

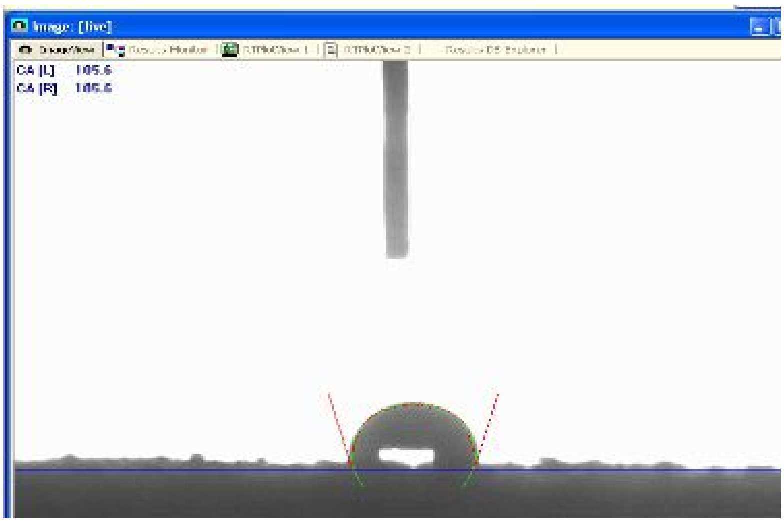

(b)

Figure S3: Sandstone rock tablets coated with oil and after the final treatment with nonylphenol alcoholic micellar systems (SMA-NP) used for the wettability test.

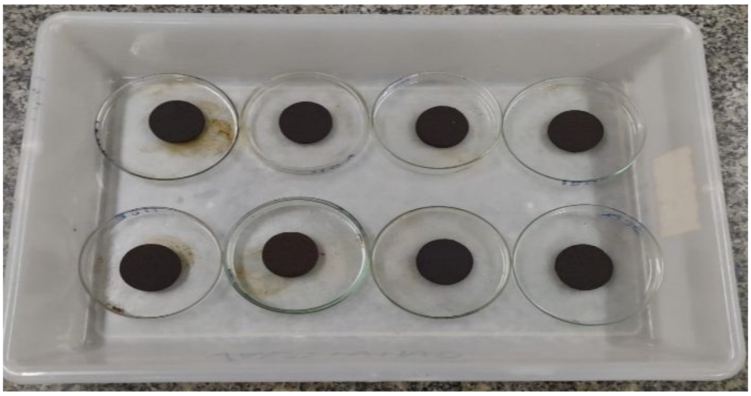

The samples to be tested were fixed on the test platform of a Kruss goniometer, and the syringe was installed. The needle position and droplet shape were controlled by the control panel; contact angles were measured, and the final result was read by the instrument.

Figure S4: Pareto chart for the studied variables with 95% confidence for the AMS: (a) NP-9.5EO, (b) NP-11EO, and (c) NP-15EO.

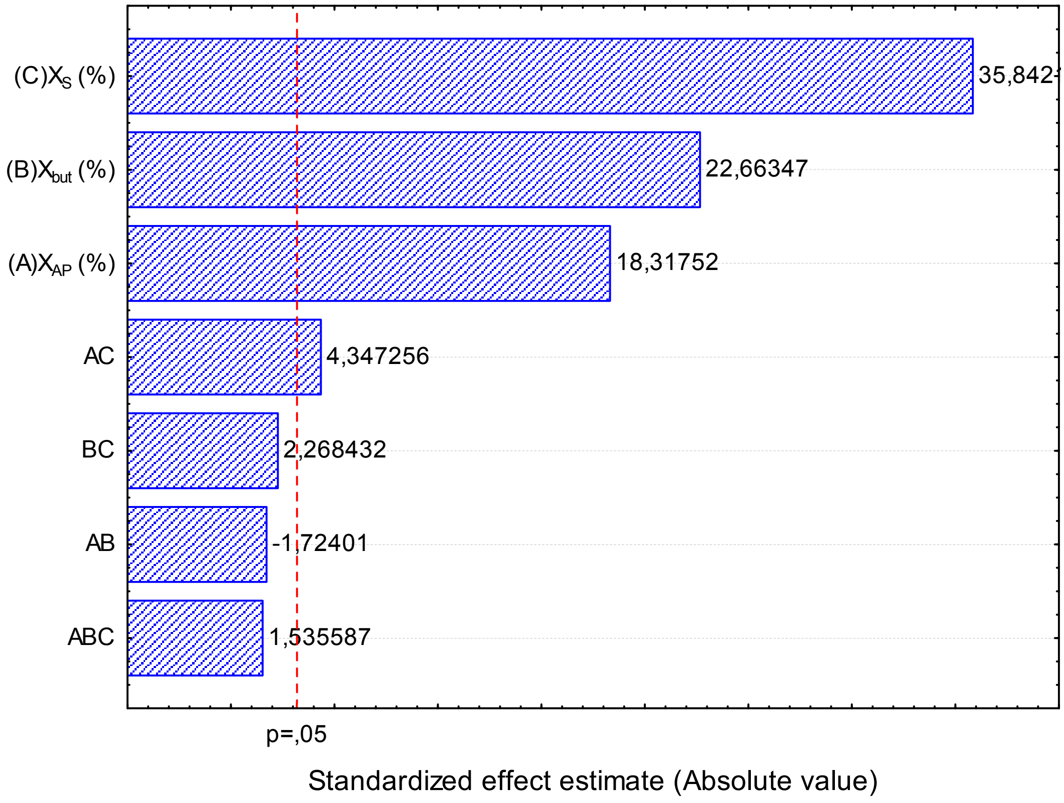

(a)

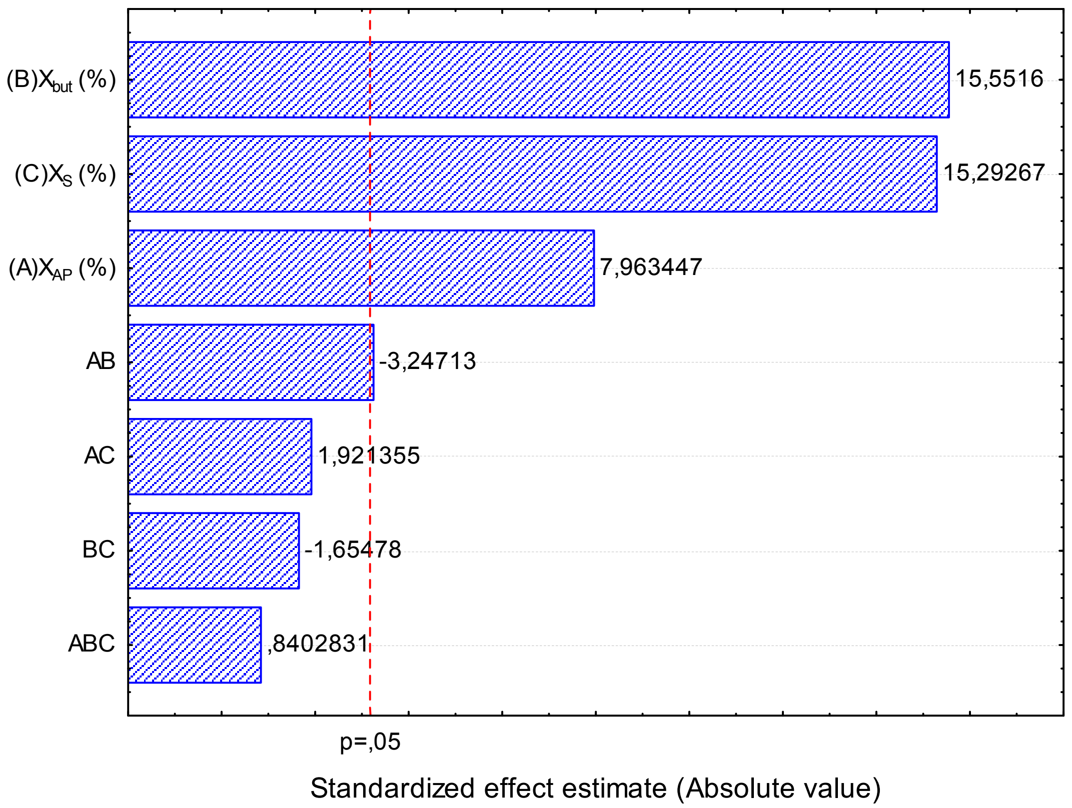
(B)X_{but} (%)
(C)X_S (%)
(A)X_{AP} (%)
AB
AC
BC
ABC
15,5516
15,29267
7,963447
-3,24713
1,921355
-1,65478
,8402831
p=,05
Standardized effect estimate (Absolute value)

(b)

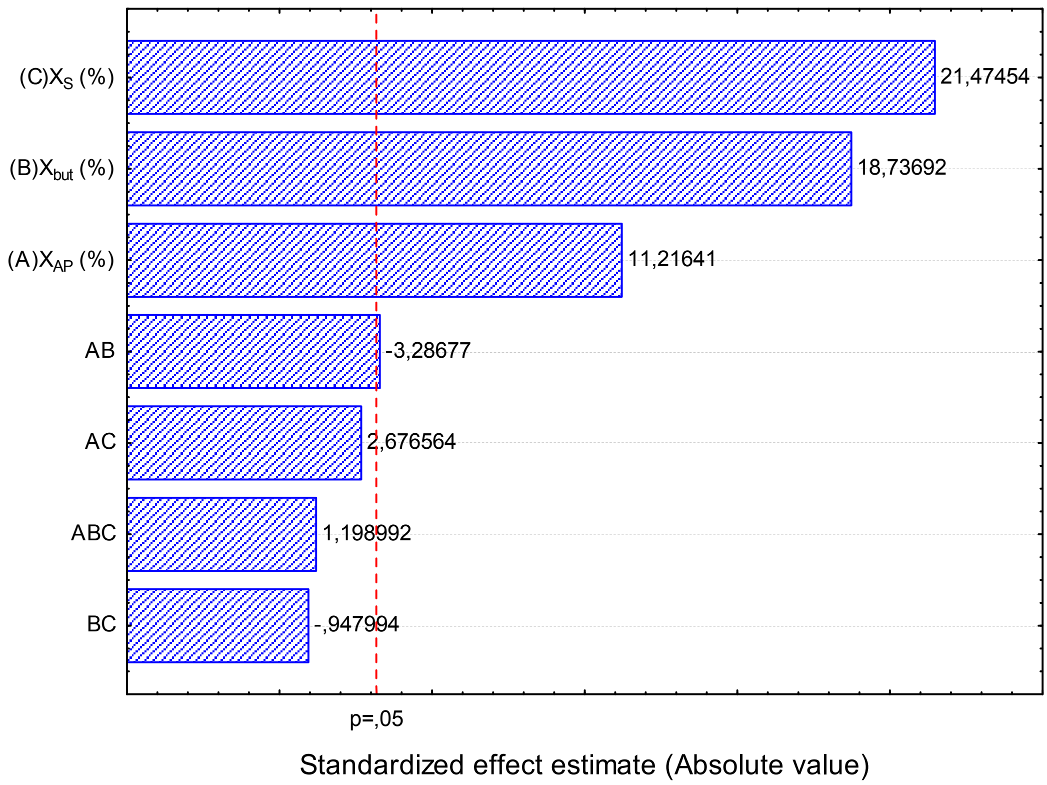
(C)X_S (%)
(B)X_{but} (%)
(A)X_{AP} (%)
AB
AC
ABC
BC
21,47454
18,73692
11,21641
-3,28677
2,676564
1,198992
-,947994
p=,05
Standardized effect estimate (Absolute value)

(c)

Figure S5: Graphs of predicted values by the equation versus observed values for the AMS: (a) NP-9.5EO, (b) NP-11EO, and (c) NP-15EO.

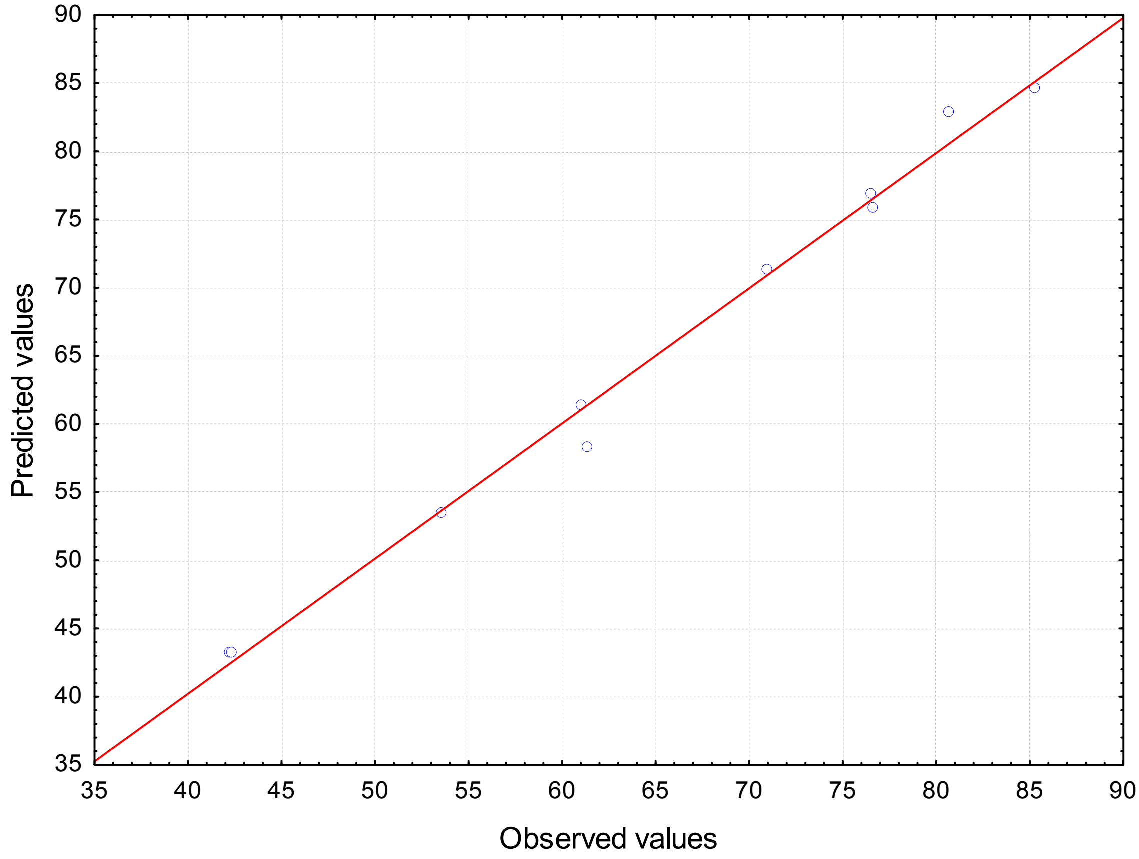

(a)

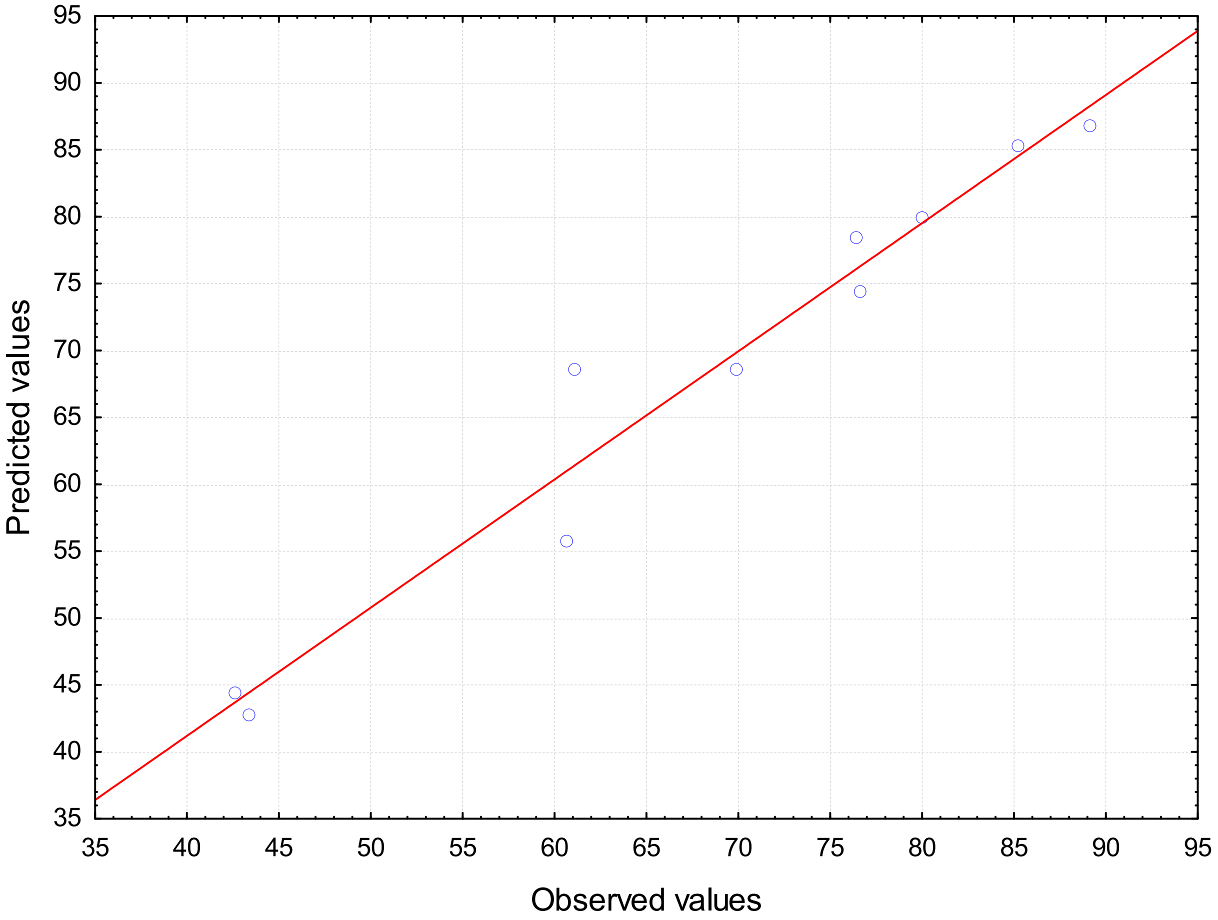

95
90
85
80
75
70
65
60
55
50
45
40
35
Predicted values
35 40 45 50 55 60 65 70 75 80 85 90 95
Observed values

(b)

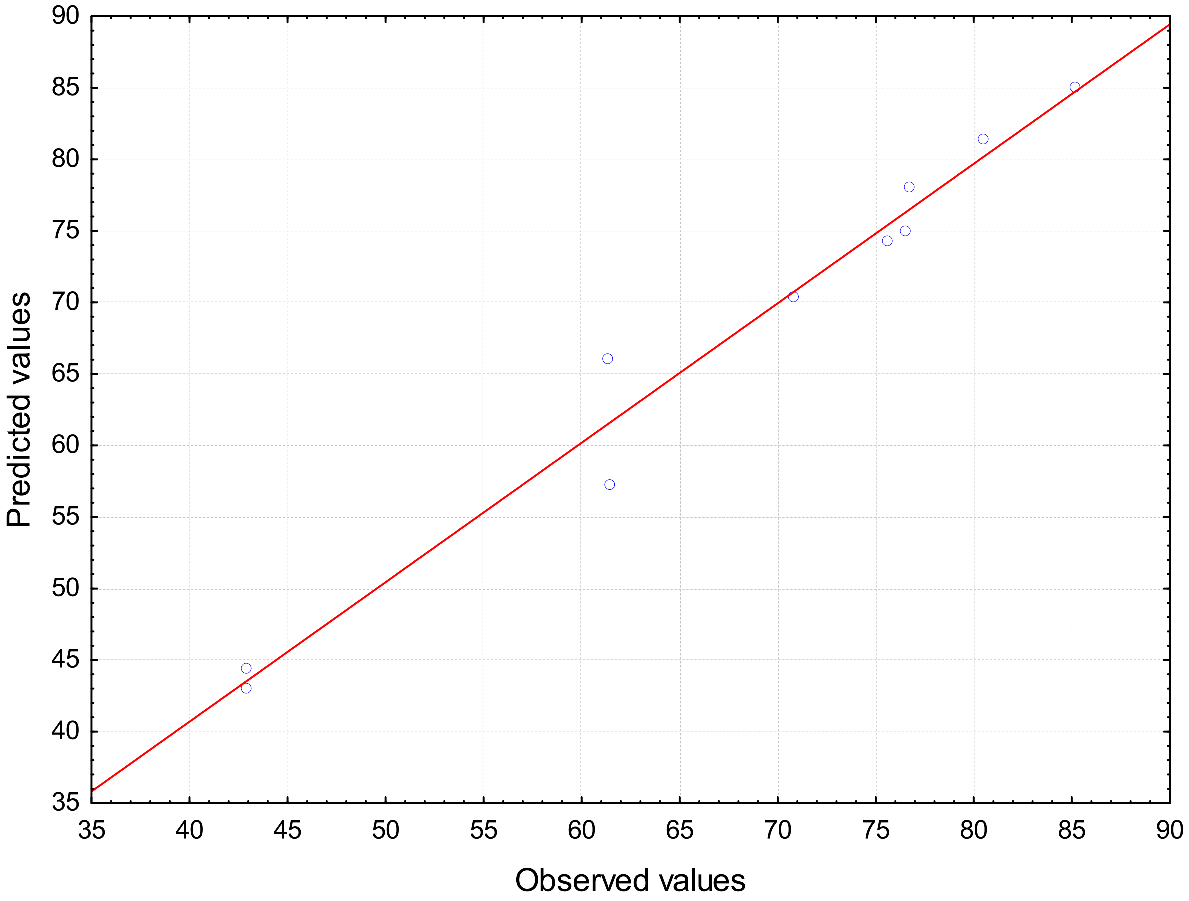

90
85
80
75
70
65
60
55
50
45
40
35
Predicted values
35 40 45 50 55 60 65 70 75 80 85 90
Observed values

(c)

Figure S6: Response surface graphs for the adsorption efficiency of (a) AMS NP-9.5EO, (b) AMS NP-11EO, and (c) AMS NP-15EO within the Scheffé liquid domain.

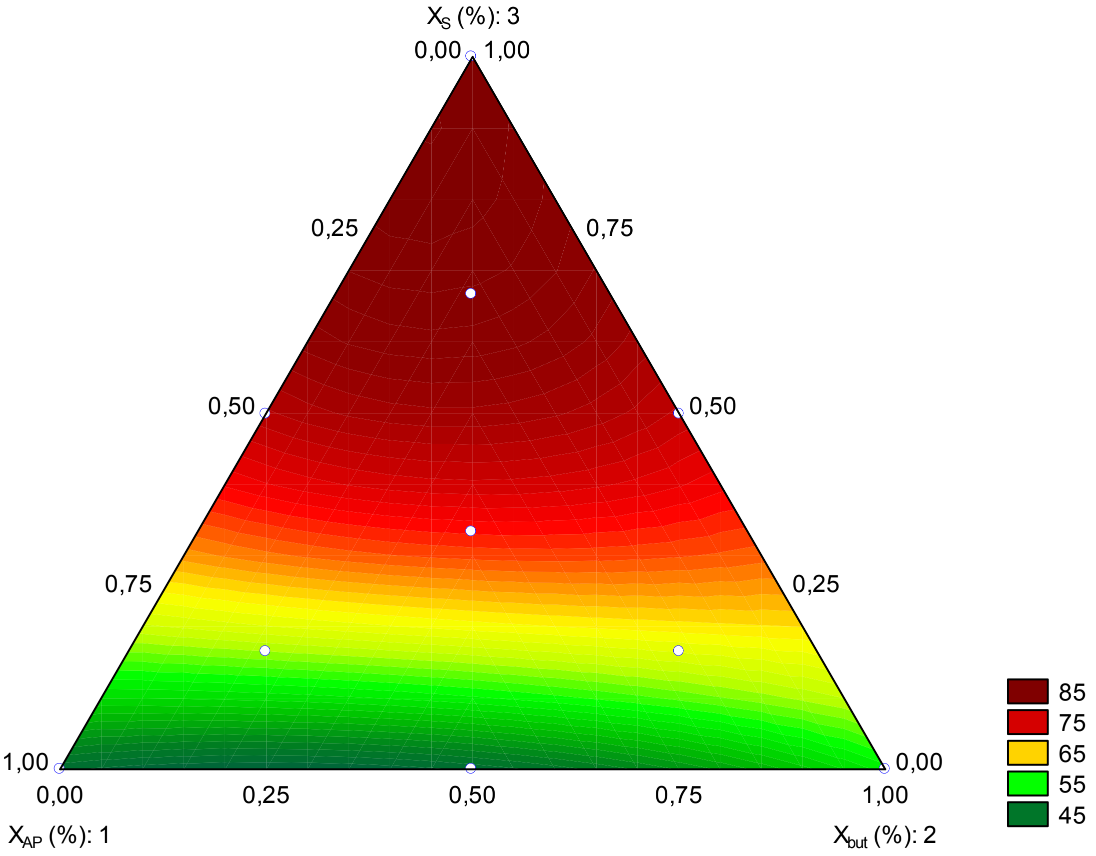

(a)

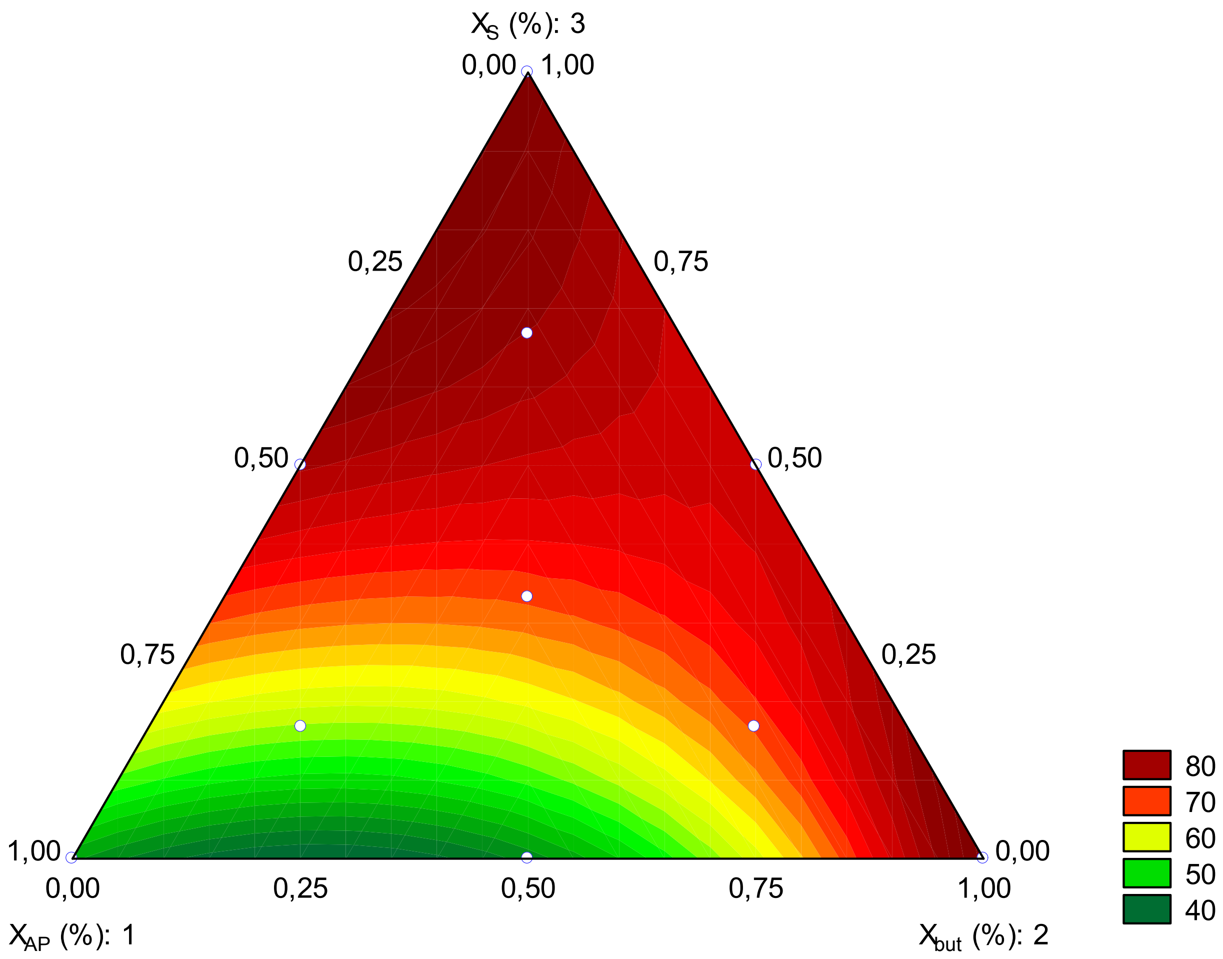

X_S (%): 3
0,00
1,00
0,25
0,75
0,50
0,50
0,75
0,25
1,00
0,00
0,00
0,25
0,50
0,75
1,00
X_AP (%): 1
X_but (%): 2
80
70
60
50
40

(b)

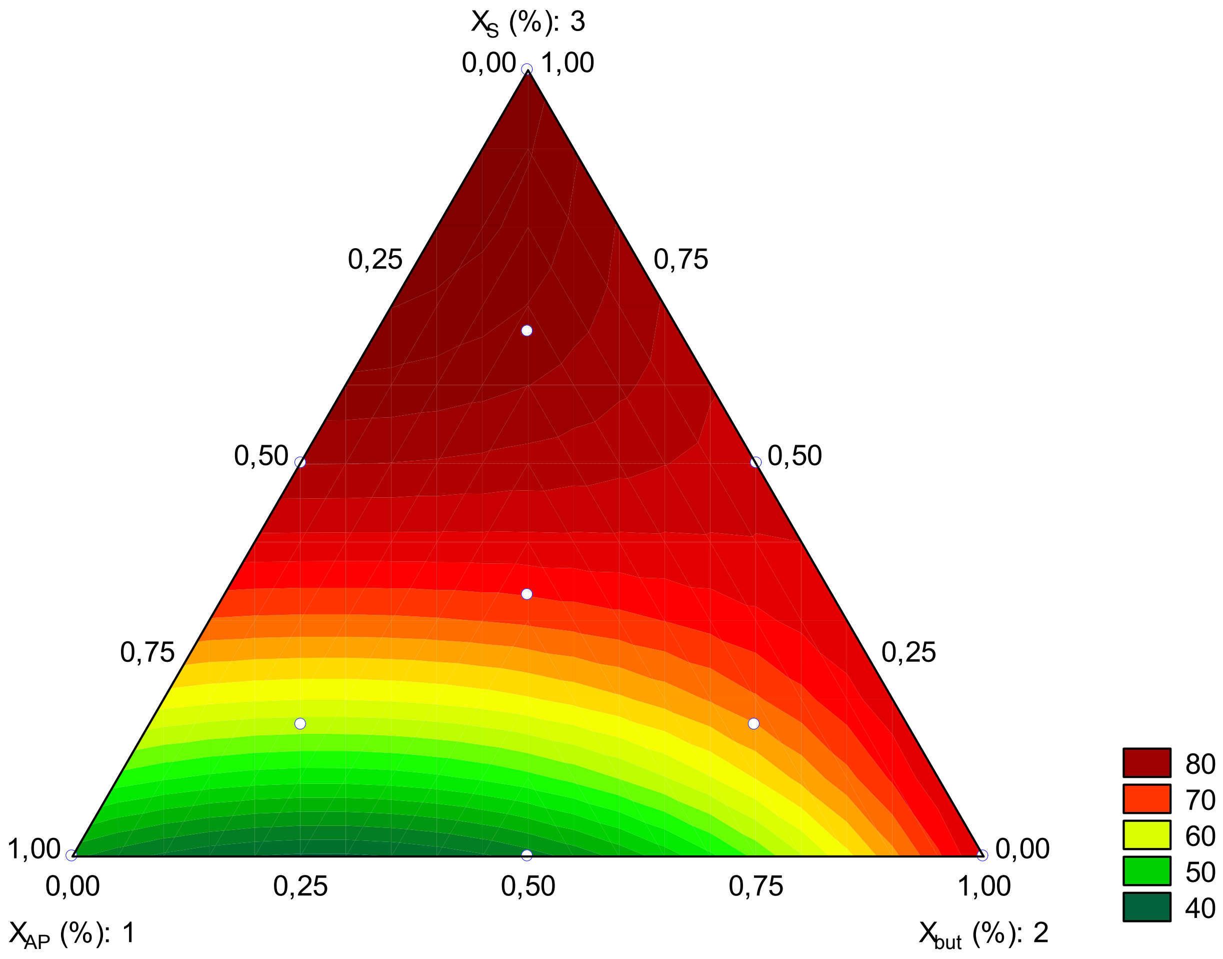

(c)

Upon analyzing the response surfaces, it is noted that the best adsorption efficiencies are achieved at high surfactant values and low butanol concentration, meaning these two parameters are inversely related. In Figure S6a, the best results for adsorption efficiency are achieved with high surfactant concentration values, while in Figures S6b and S6c, it can be observed that even with the decrease in the amount of surfactants, the systems did not undergo a significant decrease in adsorption efficiency.

Table S3: Parameters obtained from the adsorption isotherm models at 40°C for nonylphenols NP-9.5EO, NP-11EO, and NP-15EO.

Surfactant	Langmuir			
Surfactant	q_m $(mg.g^{1})$	$K_L(L.g^{-1})$		R^2
NP-9.5EO	80.52	0.596		0.975
NP-11EO	68.93	0.551		0.961
NP-15EO	68.19	0.458		0.953
Surfactant	Freundlich			
	K_F $(L.g^{-1})$	1/n (-)		R^2
NP-9.5EO	60.26	0.327		0.879
NP-11EO	53.41	0.317		0.833
NP-15EO	53.29	0.296		0.814
Surfactant	Redlich-Peterson			
	K_R (L.g-1)	αR $(L.mg^{-1})β$	β	R^2
NP-9.5EO	9.55 x103	3.27 x102	1.08	0.997
NP-11EO	9.22 x103	3.26 x102	1.15	0.972
NP-15EO	9.20 x103	3.09 x102	1.16	0.985
Surfactant	Temkin			
	qT $(mg.g^{-1})$	K_T $(L.g^{-1})$		R^2
NP-9.5EO	1.66	55.27		0.964
NP-11EO	1.42	51.75		0.942
NP-15EO	1.39	46.50		0.943

Table S4: Contact angle measurements between the sandstone and titration with distilled water before and after treatment with AMS and percentage reduction compared to untreated rock.

Untreated tablets	Contact angle (Θ)	Tablets with treatment	Contact angle (Θ)	%
sandstone rock	105.6°	SMA NP 9.5EO	13.5°	87.22
		SMA NP 11EO	18.8°	82.00
		SMA NP 15EO	49.3°	53.31

Analyzing the results from Table S4, it is observed that the untreated sandstone, with its surface coated with oil, behaved as oil-wet upon receiving a drop of distilled water, meaning it exhibited a hydrophobic surface with a high contact angle of 105.6°, remaining constant, which hinders oil flow in the production process.

Observing the behavior for tablets treated, it is noted that upon contact with the drop of distilled water, all systems were able to significantly reduce the contact angle. The AMS NP 9.5EO reduced the angle to 13.5°, representing a reduction of approximately 87.22%; AMS NP 11EO reduced it to 18.8°, a reduction of 82%; and AMS NP 15EO reduced it to 49.3°, a reduction of 53.31%. Hence, this study proved that treating Botucatu sandstone with alcoholic micellar systems composed of nonylphenols was effective in reversing its wettability, showing a significant reduction in the contact angle.

Figure S7: Diffraction patterns of the Botucatu sandstone rock before and after adsorption.

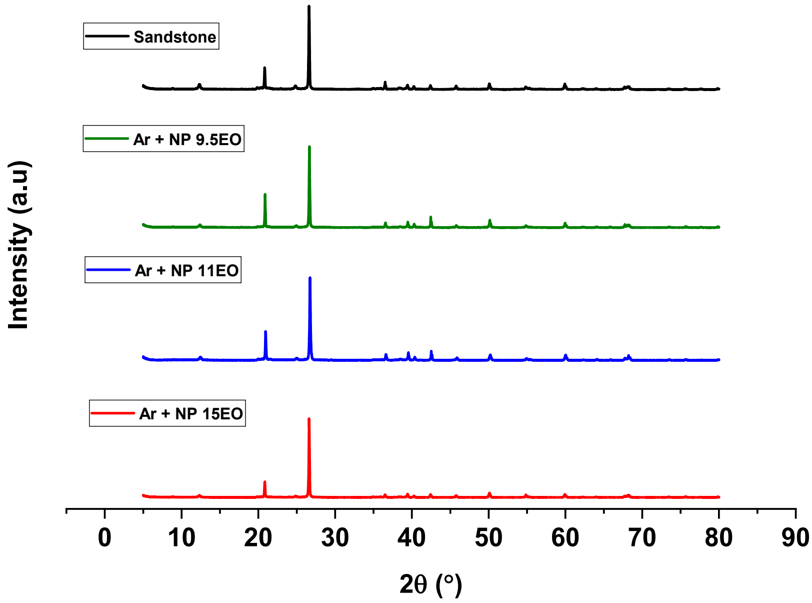

FTIR measurements were conducted for nonylphenols and for the different sandstone samples before and after the adsorption experiments. The main objective of this technique was to detect the different structures of chemical species and provide qualitative measurements based on the absorption and vibrational bands of NP-9.5EO, NP-11EO, and NP-15EO.

Figure S8 shows the FTIR spectra for the nonylphenols, while Figure S8 (a) shows for the pure sandstone and Figure S8 (b-d) shows for the sandstone adsorbed with AMS NP-9.5EO, NP-11EO, and NP-15EO, respectively.

Figure FS8: FTIR spectrum for (a) pure sandstone, (b) sandstone adsorbed with AMS NP-9.5EO, (c) sandstone adsorbed with AMS NP-11EO, and (d) sandstone adsorbed with AMS NP-15EO.

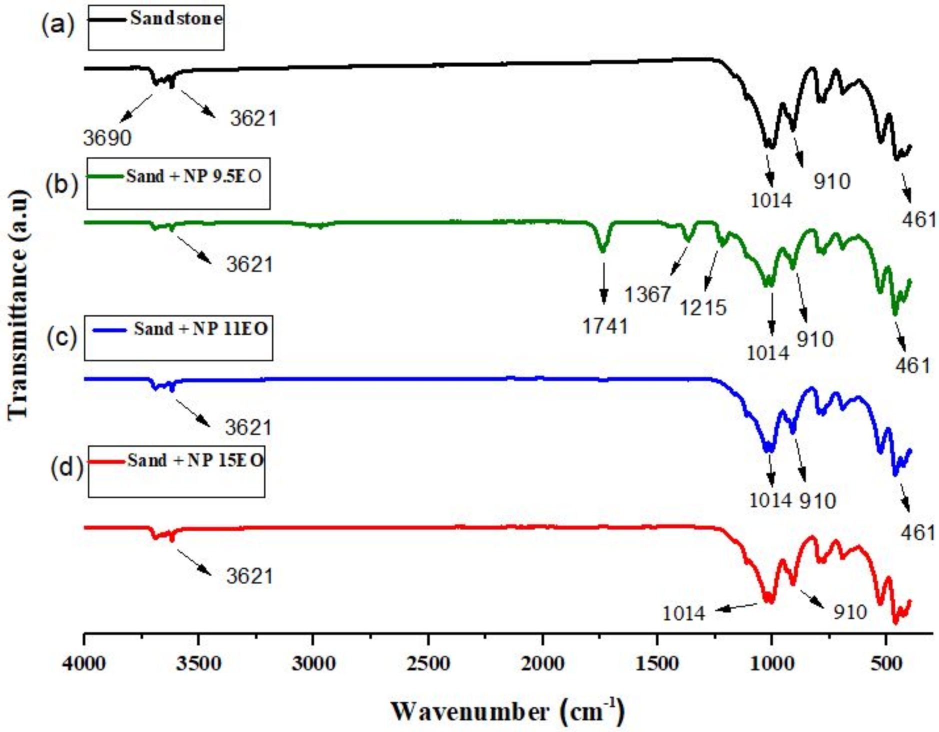

As observed in the Figures above, the FTIR spectrum of pure sandstone exhibits bands at 461 cm^{-1} and 1014 cm^{-1} corresponding to the vibrational stretching region for the symmetric (υSi-Os) and asymmetric (υSi-Oas) Si-O groups, respectively, indicating that the sandstone contains pure silica in its main composition. Additionally, bands at 910 cm^{-1} corresponding to quartz grains are observed. The sandstone sample also shows bands at 3621 cm^{-1} to 3690 cm^{-1} corresponding to the symmetric (υC-Hs) and asymmetric (υC-Has) stretching of the -CH_2 groups, respectively.

After the adsorption of the surfactants, a slight reduction in the bands at 3690-3621 cm^{-1} was observed in all spectra. A band at 1741 cm^{-1} was observed in the absorption spectrum of Figure S8 (b) of the sandstone + NP-9.5EO, likely originating from the vibrational stretching of the OH group of water molecules during the surfactant adsorption.

Figure S9: FTIR Spectrum for the nonylphenols: NP-9.5EO, NP-11EO, and 15EO.

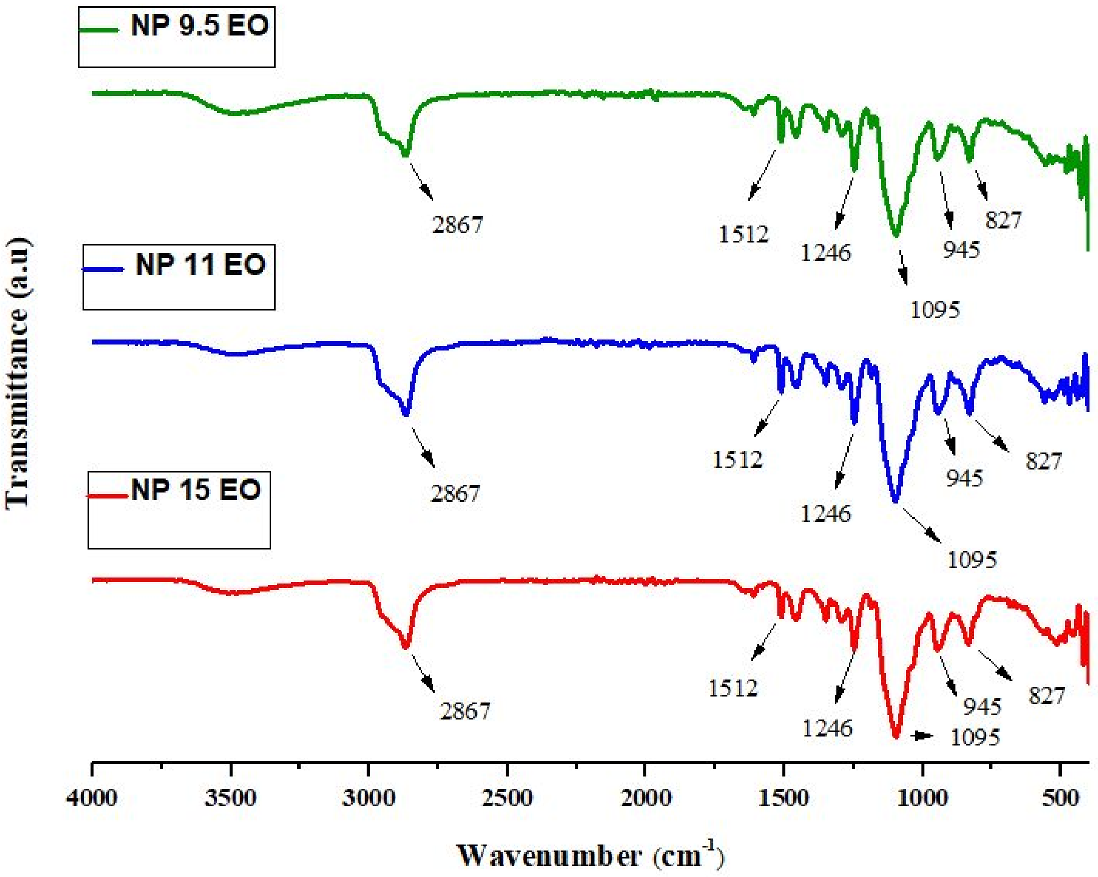

The Figure above shows absorption bands characteristic of surfactants and their respective functional groups. According to these infrared spectra, it is observed that alkylphenol ethoxylates have many functional groups containing oxygen, among which the C-O- group is the most abundant, followed by aliphatic and aromatic hydrocarbons.

The bands observed at 2960 and 2867 cm^{-1} represent the symmetric (υC-Hs) and asymmetric (υC-Has) vibrations of the CH_2 groups, respectively. Another important band is at approximately 1500-1512 cm^{-1} corresponding to the unsaturations of the C=C bonds in the aromatic ring. The bands in the range of 1200 cm^{-1} to 1095 cm^{-1} refer to the stretching of the C-O- bond, thus confirming the alcohol, methylene, aromatic, and ether groups of nonionic surfactants.

Figure S10 below shows the thermogravimetric curves of the samples studied: pure sandstone, Ar + AMS NP 9.5EO, Ar + AMS NP 11EO, and Ar + AMS NP 15EO, which relate the mass loss (%) and temperature (°C).

Figure S10: Thermal stability analysis of sandstone rock before and after adsorption with AMS (a) TGA showing the degradation or mass loss of pure sandstone, (b) Ar + AMS NP 9.5EO, (c) Ar + AMS NP 11EO, and (d) Ar + AMS NP 15EO.

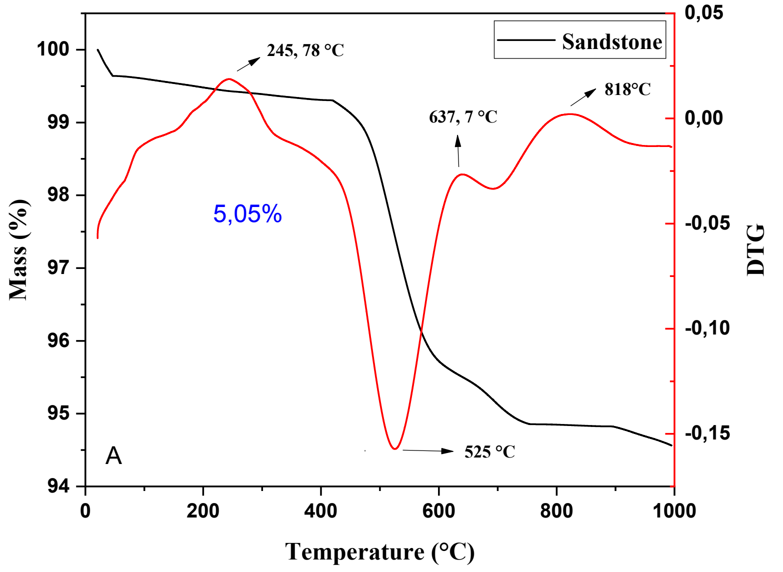

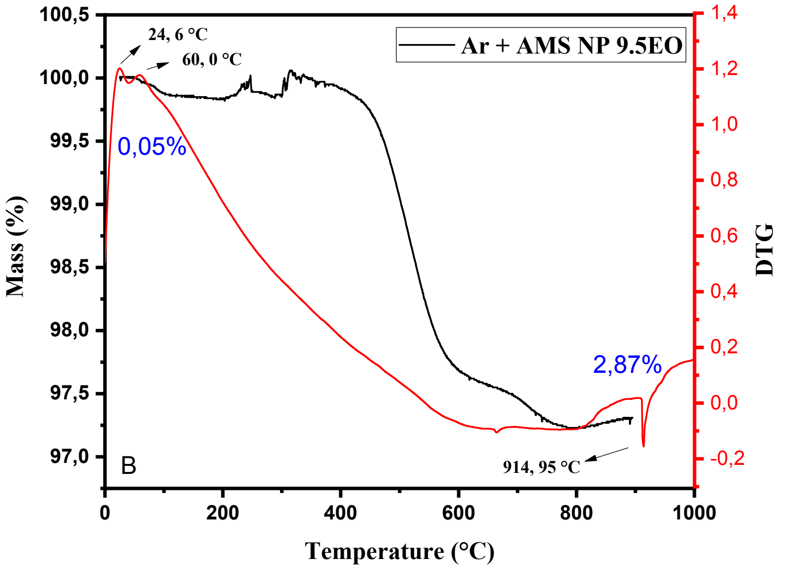

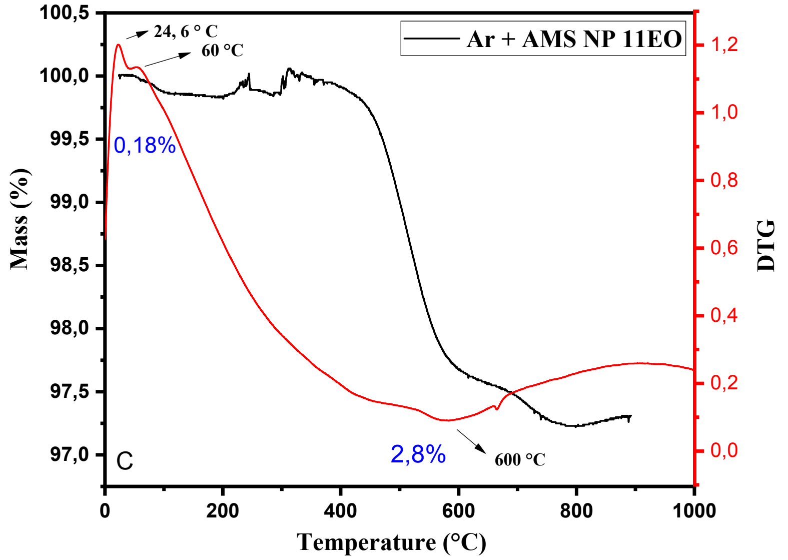

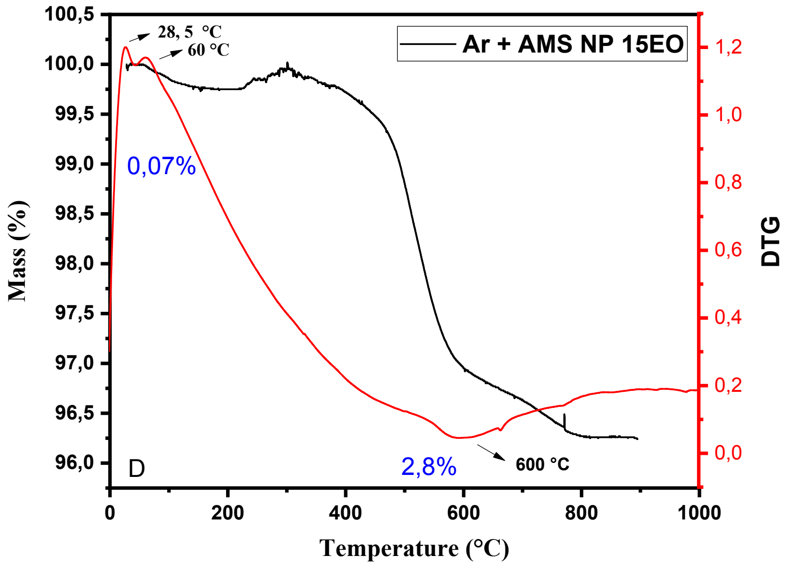

For all samples, the presence of peaks was observed as the temperature increased from 24°C to 400°C, corresponding to the release of physically adsorbed water in the pores and on the surfaces of the samples. This process resulted in a mass reduction of 5.05%, 0.05%, 0.18%, and 0.07% for pure sandstone, Sandstone + AMS NP 9.5EO, Sandstone + AMS NP 11EO, and Sandstone + AMS NP 15EO, respectively.

It is also noted that the dehydroxylation of kaolinite occurs for pure sandstone as the temperature increases from 400°C to 600°C. This is indicated by a significant sample weight loss of 5.05% and an exothermic peak in heat flow at around 525°C. As the temperature rises, two endothermic peaks occur at approximately 637.7°C and 818°C. These latter steps may be related to the recrystallization of the sample to form mullite.

Thermogravimetric analyses for sandstone treated with AMS showed greater thermal stability compared to untreated sandstone. It is observed that these thermograms did not undergo significant mass losses at temperatures from 50°C to 60°C, which are typically reached in oil wells, thus they can be used for oil recovery tests without compromising adsorption studies.

A similar behavior is observed for the three AMS-treated samples, with a mass loss of approximately 2.8%. This mass loss between 200°C and 600°C corresponds to the loss of elements present in the active chemical structure of the surfactants, while above this temperature, it can be attributed to dehydroxylation and carbon dioxide produced after the decomposition of nonylphenols.

Figure S11 shows the morphology of the rock surface through SEM of the sandstone samples before and after static adsorption experiments.

Figure S11: (a) Images of pre-adsorption and (b) Images of post-adsorption for sandstone treated with SMA NP 9.5EO, NP 11EO, and NP 15EO.

Treatment	(a) Pre-adsorption	(b) Post-adsorption
NP 9.5EO	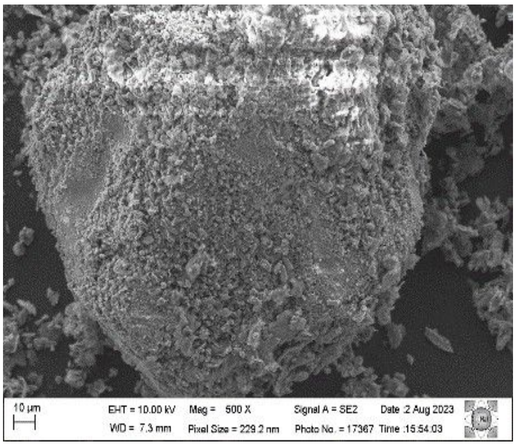	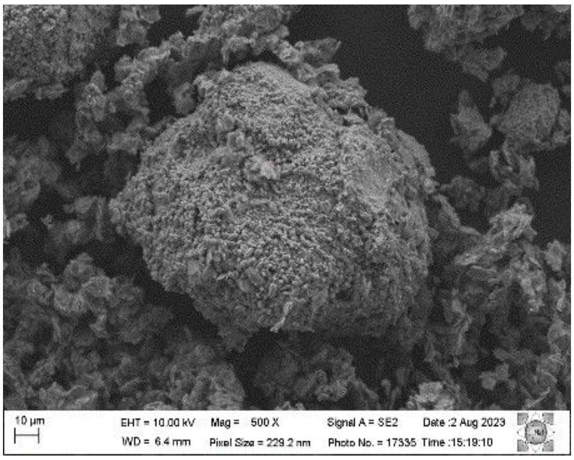
NP 11EO	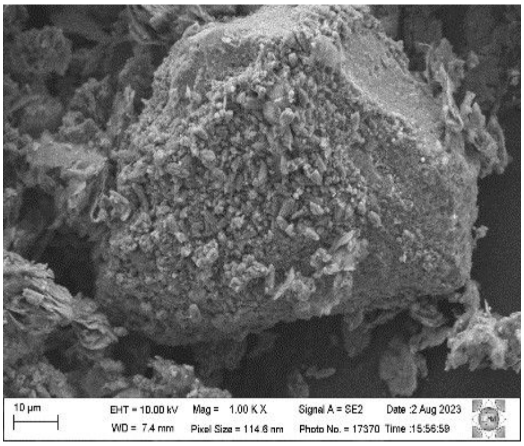	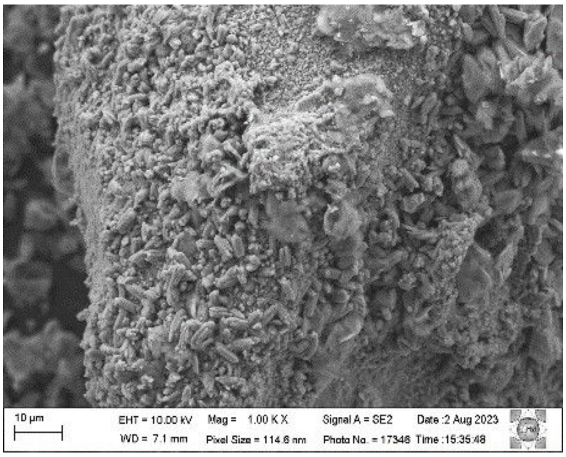
NP 15EO	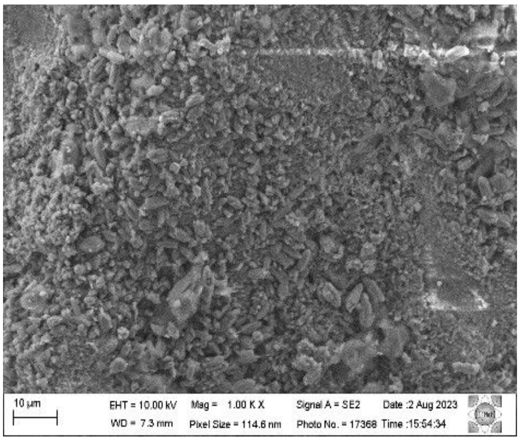	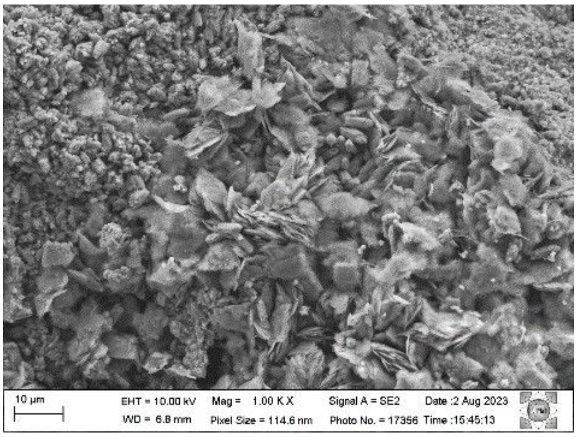

This analysis allows the observation of the enlarged region on the surface of the adsorbent, but it cannot cover the entire surface of the particles present in the samples. Therefore, locations were selected that presented particles of similar dimensions and shapes to investigate any potential changes in surface morphology caused by the static adsorption process. Figure S11 (a) shows the observations of pre-adsorption, and Figure S11 (b) shows post-adsorption.

It is observed that quartz crystals are predominantly present amidst the rough surface. Prismatic or tabular crystals of various sizes are observed, which, upon estimating the approximate chemical composition (using the EDS technique coupled with SEM), exhibit a high content of silicon and aluminum.

All sandstone samples exhibit a lamellar and rough surface before adsorption. However, after adsorption, the appearance of flakes suggests that nonylphenols were adsorbed on the surface of the rock. This slight alteration in morphology is accompanied by a decrease in surfactant concentration in the solution. The results of finite bath adsorption in this research also showed that maximum adsorptions occurred only for AMS NP 9.5EO and NP 11EO.

Figures S12 (a-d) present the EDS spectra (Energy Dispersive Spectroscopy) of the natural rock samples and those treated with nonylphenols previously analyzed by scanning electron microscopy. Through these spectra, the chemical elements present in the samples are identified.

Figure S12: EDS spectrum (a) of natural sandstone, (b) of sandstone treated with AMS NP 9.5 EO, (c) treated with AMS NP 11 EO, and (d) treated with AMS NP 15 EO.

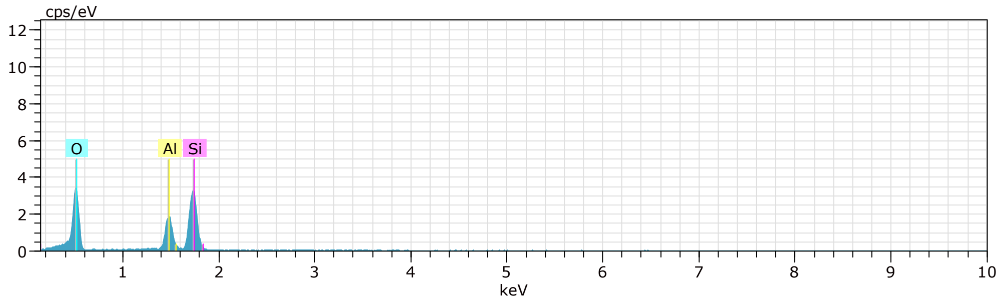

(a)

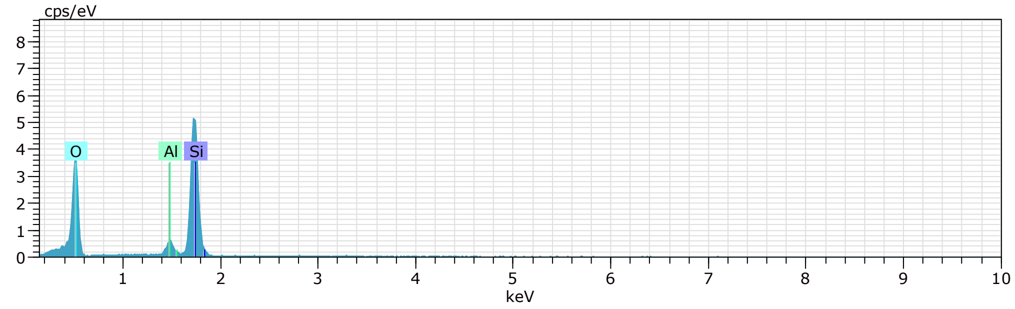

(b)

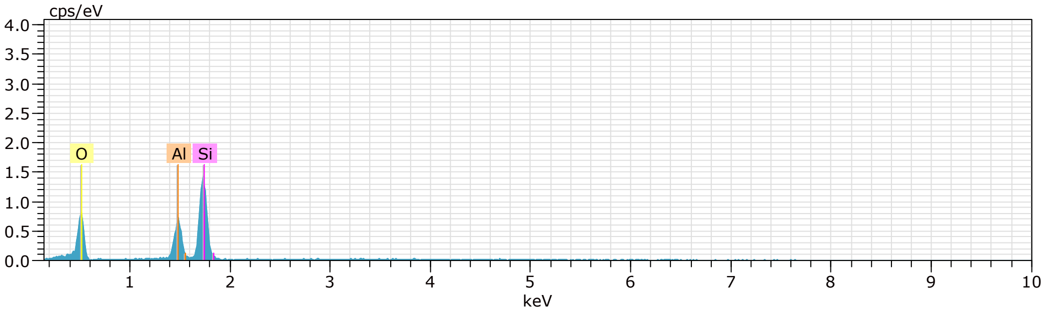

(c)

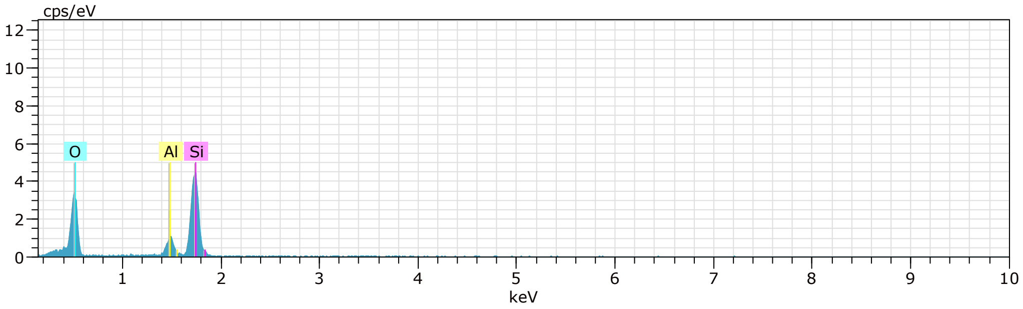

(d)

The peaks identified in the samples are characteristic of sandstone rocks, which have silicon, aluminum, and oxygen as their main elements in their chemical formula.
